# Bacterial Community Composition and Extracellular Enzyme Activity in Temperate Streambed Sediment during Drying and Rewetting

**DOI:** 10.1371/journal.pone.0083365

**Published:** 2013-12-27

**Authors:** Elisabeth Pohlon, Adriana Ochoa Fandino, Jürgen Marxsen

**Affiliations:** 1 Institut für Allgemeine und Spezielle Zoologie, Tierökologie, Justus-Liebig-Universität Gießen, Heinrich-Buff-Ring 26, Gießen, Germany; 2 Limnologische Flussstation des Max-Planck-Instituts für Limnologie, Schlitz, Germany; Argonne National Laboratory, United States of America

## Abstract

Droughts are among the most important disturbance events for stream ecosystems; they not only affect stream hydrology but also the stream biota. Although desiccation of streams is common in Mediterranean regions, phases of dryness in headwaters have been observed more often and for longer periods in extended temperate regions, including Central Europe, reflecting global climate change and enhanced water withdrawal. The effects of desiccation and rewetting on the bacterial community composition and extracellular enzyme activity, a key process in the carbon flow of streams and rivers, were investigated in a typical Central European stream, the Breitenbach (Hesse, Germany). Wet streambed sediment is an important habitat in streams. It was sampled and exposed in the laboratory to different drying scenarios (fast, intermediate, slow) for 13 weeks, followed by rewetting of the sediment from the fast drying scenario via a sediment core perfusion technique for 2 weeks. Bacterial community structure was analyzed using CARD-FISH and TGGE, and extracellular enzyme activity was assessed using fluorogenic model substrates. During desiccation the bacterial community composition shifted toward composition in soil, exhibiting increasing proportions of *Actinobacteria* and *Alphaproteobacteria* and decreasing proportions of *Bacteroidetes* and *Betaproteobacteria*. Simultaneously the activities of extracellular enzymes decreased, most pronounced with aminopeptidases and less pronounced with enzymes involved in the degradation of polymeric carbohydrates. After rewetting, the general ecosystem functioning, with respect to extracellular enzyme activity, recovered after 10 to 14 days. However, the bacterial community composition had not yet achieved its original composition as in unaffected sediments within this time. Thus, whether the bacterial community eventually recovers completely after these events remains unknown. Perhaps this community undergoes permanent changes, especially after harsh desiccation, followed by loss of the specialized functions of specific groups of bacteria.

## Introduction

Stream ecosystems in many regions of the world, including temperate zones, experience increasing hydrological disturbances, reflecting global climate change, with more frequent and longer lasting dry periods and more frequent and severe floods [1], [2], [3]. Several studies on the effects of stream desiccation during extended periods of the year have been performed for the Mediterranean [4], [5], [6]. However, the streams and rivers in temperate regions are also becoming affected by longer lasting droughts [2], [7], [8], which in many cases can be attributed to both climate change and enhanced human water withdrawal [9]. Local climate models for the German state of Hesse predict a decrease in the summer runoff of up to 15% [10], consistent with predictions for other European regions [11].

Sediments in small streams are colonized by bacteria with high densities, providing nearly 70% of heterotrophic production and utilizing macromolecular organic matter via the excretion of extracellular enzymes and are thus the dominant habitats for carbon turnover in streams [12]. The decomposition of organic matter through microorganisms is reduced after stream fragmentation during the initial development of droughts [5], [13], and the input from bioavailable dissolved organic carbon is reduced [14]. The activity of extracellular enzymes located at the base of the microbial food chain represents a critical step in organic carbon turnover for the entire stream ecosystem [15]. The potential activity of extracellular enzymes is reduced during and after droughts [16] but withstands 3 months of desiccation [17]. As shown in a pilot study on Mediterranean and temperate streambed sediments, the enzyme activity returns to initial levels occurring in wet sediments within 4 days of rewetting, but the rate of recovery is dependent on the intensity of desiccation [7].

Reduced organic carbon, nitrogen and phosphorus inputs and a decreasing organic:inorganic nutrient ratio during desiccation favor autotrophic life forms [14], [18], which in turn enhance beta-glucosidase activity [19]. Algal communities are highly affected by desiccation. For example, diatoms are found to be unable to withstand short dry periods of 15 days [20], [21]. However, viable algae, which resist weeks of desiccation stress, are the basis for the recovery of the algal community during the rewetting process [22]. Sediment desiccation not only decreases several microbial functions, such as EEA (extracellular enzyme activity), but also reduces biomass and drastically changes the community structure [5], [23], [24]. The general functionality of the community [4], including biogeochemical processes, such as carbon and nitrogen turnover, are also affected [25], [26]. These changes can be particularly significant for stream ecosystems if the structure and function is changed over a long time period.

The phylogenetic composition of bacterial communities in streams primarily comprises *Alpha-, Beta-* and *Gammaproteobacteria, Bacteroidetes, Actinobacteria, Acidobacteria* and *Firmicutes* [27], [28], [29]. The communities in stream water are considerably distinct from sediment communities, although the flowing water habitat of streams typically does not offer enough residence time for the development of specific communities [29]. Thus, this difference remains an unexplained paradox [30]. In temporary Mediterranean streams, *Alpha-* and *Betaproteobacteria* increase [4], [5] during desiccation events, and the bacterial communities contain higher proportions of Gram-positive bacteria [7]. Bacteria belonging to the Gram-positive phyla of *Actinobacteria* and *Firmicutes* are not as vulnerable as Gram-negative bacteria to disruption through osmotic stress and drought [23], [31]. The intensity of the community shift is dependent on the level and duration of desiccation and is more distinct after longer droughts [7]. The recovery of streambed sediment communities after desiccation might occur through the development of microorganisms to generate adaptation strategies for desiccation [32] and thus might result in the occurrence of higher proportions of groups known from terrestrial ecosystems, even after the rewetting of sediments.

Microbial processes respond differently to varying durations of drying periods. Partial drying of streams decrease phosphorus and nitrogen availability, whereas complete desiccation diminishes microbial activity and inhibits anaerobic bacterial processes, such as denitrification [33]. For example, after a short desiccation event of approximately 3 days, both nitrification and denitrification were decreased in a Kansas stream [34]. In contrast, drought also stimulated nitrification, whereas denitrification was inhibited [35].

The potential problems of increasing desiccation events in temperate streams and rivers have been neglected. The impact on stream structure and function is obviously of increasing importance in temperate regions. In the present study, sediment obtained from the Breitenbach, a small unpolluted stream in eastern Hesse (Germany), was dried in the laboratory for 13 weeks and then rewetted for 2 weeks. The Breitenbach was the object of a long-term ecosystem study (from 1969–2006) at the Limnologische Flussstation in Schlitz (Hesse), a division of the former Max Planck Institute for Limnology. Thus, profound background data for studies on stream ecology are available from this site [36]. The stream was exposed to increasing phases of drought within the last decades, thereby representing a suitable model environment to study desiccation effects in stream ecosystems [37]. The development of the bacterial community and microbial extracellular enzyme activity was assessed in the artificially dried and rewetted sediment. Three different drought scenarios (fast, intermediate, and slow) were simulated over the 13-week period, followed by rewetting of the fast desiccated sediment using a perfused core technique [15]. The origin of the bacterial community during recovery is unknown; thus, the rewetting experiment was divided into two groups. One group was exposed to original unaffected stream water from the Breitenbach, whereas the second group was perfused with sterile water from this stream.

For the desiccation experiment, we hypothesized that (i) the structure of the bacterial community changes depending on the duration and intensity of drying, (ii) the bacterial community-coupled carbon turnover decreases according to the length of the drought and (iii) the extracellular enzymes might withstand drought and remain potentially active. For the rewetting experiment, we assumed that (iv) extracellular enzyme activity recovers within 14 days of rewetting and (v) the development of the community structure differs with the type of water used for rewetting.

## Materials and Methods

### Study Site

The study was conducted using sediments from the Breitenbach, a first-order Central European upland stream (9°39′E, 51°39′N). It originates 350 m a.s.l. and enters the River Fulda 4,200 m downstream at 220 m a.s.l. (for details: [12], [38], [39]). During the 1970s and 1980s, the upper reach of the stream dried for several weeks during autumn [40]. However, in the decades since 1990, the desiccation of the upper reach occurred more frequently and for longer times, reflecting decreasing precipitation and intensified water withdrawal, occasionally showing no discharge for the entire year [37].

The stream and nearly the whole catchment area was dedicated as a nature reserve area in 1993 to enable scientific studies on a typical stream ecosystem [39]. The research permit for the present study was obtained from the Obere Naturschutzbehörde in Kassel (Germany).

### Experimental Setup

Sandy sediment was sampled from the streambed surface (0–3 cm depth) in the middle reach of the Breitenbach on June 6^th^, 2010 and brought to the laboratory within two hours. As the sediment was not completely homogenous, the sample was cautiously blended (avoiding the destruction of fragmentary biofilm structures with dispersed patches of microorganisms on the sediment grains) to achieve a homogeneous feedstock. A total of 150 mL per replicate was added to 250-mL polypropylene containers achieving a sediment height of approximately 7 cm. The grain size distributions were <0.063 mm, 3.4%; 0.063–0.2 mm, 10.9%; 0.2–0.63 mm, 71.8% and 0.63–2.0 mm, 13.9% (per weight percentages).

Three different approaches were used to simulate different desiccation scenarios. To simulate slow droughts, the containers were covered with plastic foil. Fast desiccation was achieved after uncovering the sediment containers, whereas covering the sediment with gauze (40 µm mesh size) simulated intermediate desiccation. A total of 20 replicates per scenario were stored at 20°C in a climatic chamber in the dark. Analyses of the samples, with 4–5 containers, were performed on the wet sediment upon initial sampling and after 2, 4, 8 and 13 weeks of desiccation. To complete the suite of analyses, the entire sediment of one container was required.

Five additional uncovered containers (fast drying scenario) were prepared for the rewetting experiment, initiated after 13 weeks of desiccation using a perfused core technique. This technique simulates the natural process occurring in many streams, including the Breitenbach, whereby groundwater enters the stream by diffuse perfusion through the streambed. This technique had been successfully used to examine the development of microbial community composition and measure microbial community metabolism, even in long-term experiments of up to 6 months [15], [41], [42], [43]. The perfused core technique was also established as a suitable tool for evaluating the recovery of microbial communities during rewetting of desiccated streambed sediments [7], [17], [37]. To initiate rewetting, dry sediment was filled into polypropylene syringes with previously removed upper ends, achieving cores of 2.0 cm in depth and 2.0 cm in diameter. The syringes were sealed with silicone rubber stoppers, each incorporating a 2 mm i.d. glass tube, and placed in a temperature-regulated incubator in the dark, followed by perfusion at 12°C from below with stream water at a perfusion velocity of 2.0 mL cm^−2^ h^−1^. This velocity corresponded to the natural diffuse water-inflow velocity through the streambed at a low water level in the Breitenbach.

The EEA was measured from water leaving the cores on top, facilitating the use of the same cores for the 2-week duration of the experiment. To determine the bacterial community structure, the cores were disrupted and separate cores were sampled after 1, 2, 3, 6, 10 and 14 days of rewetting. To measure EEA, the cores were perfused with stream water, filtered (0.22 µm pore size) and sterilized through tyndallization (boiling for 3 consecutive days). To determine the bacterial community structure, two sets of cores were prepared, which were perfused with either sterilized or untreated, freshly sampled Breitenbach stream water.

### Chemical Characteristics

The moisture was determined by weight loss after drying the sediment at 105°C to constant weight. The C:N ratio was estimated using the vario EL III CNS Elemental Analyzer (Elementar Analysensysteme, Hanau) according to DIN 13878 [44] and DIN 10694 [45] procedures. Nitrate and ammonium were determined photometrically through continuous discharge flow-through analysis (TRAACS 800 Autoanalyzer, Bran+Luebbe, Norderstedt) after extraction [46].

### Abundance of Prokaryotes and Bacterial Community Composition

The samples for the determination of bacterial abundance and CARD-FISH analysis (1.0 mL sediment in 5 replicates of each treatment and time step) were fixed with paraformaldehyde, sonicated and concentrated onto white polycarbonate filters (pore size 0.2 µm, GTTP, Sartorius, Göttingen, Germany) as previously described [37]. The cells attached onto the surfaces of the filters were stained using SYBR Green I solution [47] using epifluorescence microscopy [37]. The abundance of bacteria belonging to different taxonomic groups was determined through catalyzed reporter deposition fluorescence in situ hybridization (CARD-FISH) [37], [48] on other pieces of the same filters. Horseradish peroxidase-labeled probes, linked with Alexa_488_ as a fluorochromic dye and specific for *Bacteria*, *Alpha-, Beta*- and *Gammaproteobacteria* and *Actinobacteria*, *Firmicutes* and *Bacteroidetes*, were used (Table S1).

For the TGGE (temperature gradient gel electrophoresis) analysis, the sediment was stored at −20°C until DNA extraction. Amplification of bacterial 16S rRNA genes, separation of gene fragments and evaluation of gels were conducted as previously described [29]. The TGGE bands were treated as operational taxonomic units (OTUs), a surrogate for bacterial species. The relative intensities of the bands (as a measure of abundance) were calculated for each lane as the basis for further statistical treatment (XLSTAT 2008.6.01, Addinsoft SRAL, Andernach, Germany). The cluster analysis was performed, calculating Bray-Curtis dissimilarities for each pair of lanes; dendrograms were constructed using the Ward method. Correspondence analysis (CA) was used as another approach to examine the major tendencies of the variance of the bacterial community structures on the basis of TGGE profiles [49]. The Shannon-Wiener diversity index H_S_ and evenness J were calculated as previously described [7]. Due to technical difficulties (limited numbers of lanes per TGGE gel), we could not compare all samples from the desiccation and rewetting experiments within one gel run. Thus, the DNA profiles from both experiments were analyzed separately. At least one sample was used from all time steps in the desiccation scenarios, but 2 replicates were considered for the evaluation of the initial wet sediment and the 4- and 8-week desiccation steps. To evaluate the community patterns in the rewetting experiment, 2 replicates per scenario per time step were processed.

### Extracellular Enzyme Activity

#### Desiccation

The activities of alpha- and beta-glucosidase, beta-xylosidase and phosphatase were measured using artificial 4-methylumbelliferyl (MUF) substrates: MUF-α-D-glucoside, MUF-ß-D-glucoside, MUF-ß-D-xyloside and MUF-phosphate, respectively. To analyze aminopeptidase activity, leucine-MCA (L-leucine-4-methyl-coumaryl-7-amide) was used (all obtained from Sigma-Aldrich Chemie, Steinheim, Germany). Five replicates of 1 mL sediment samples were transferred to glass tubes in which 8 mL of filtered (0.2 µm pore size) and boiled stream water and artificial substrate solution were added, achieving a 0.3 mmol L^−1^ final concentration. This concentration had been previously determined to be in the saturation range of EEA. The samples were incubated for one hour at 12°C in a shaking water bath. After adding 1 mL of 0.05 M glycine buffer (pH 10.4), boiling and centrifugation, the fluorescence in the supernatant was measured using a fluorescence spectrophotometer (SFM 25, Kontron; emission and excitation wavelength 450 nm and 365 nm, respectively, for MUF and 443 and 379 nm, respectively, for MCA [50]). The quantification of the hydrolyzed substrate compounds was achieved through calibration with standard MUF and MCA solutions [51]. For further details, refer to [37].

#### Rewetting

The substrates were added at final concentrations of 0.3 mmol L^−1^ to the perfusion water (three replicate cores per substrate). Water containing the appropriate substrates was perfused through the cores reserved for each substrate at defined time intervals at the beginning and after 1, 2, 3, 6, 10 and 14 days of rewetting. In between the cores were perfused with water without substrates. Further details on the procedure are provided in [7], [15].

#### Multiple enzymatic functions

To characterize the effects of drying and rewetting on multiple enzymatic functions, we used the measurement of five extracellular enzymes as an indication of the utilization potential for the organic material. We followed a previously described logic [52] to define specific threshold levels of enzyme activities satisfactory for maintaining community functioning. When any individual enzyme activity decreases below the threshold, this specific function is assumed as lost. However, when the enzyme activity in the community performs above the threshold, this function is considered retained or recovered. Hence, we calculated the probability that the activity of all 5 enzymes in all replicates exceeded the given threshold. Two threshold levels, corresponding to 50% and 25% of the initial activity in wet sediments, were used, as a general agreement concerning a suitable threshold has not been achieved [52]. A probability value of 1 means that the activities of the extracellular enzymes in all replicates are above the threshold. A value of 0 indicates that none of the enzyme activity measurements exceed the threshold.

### Statistical Analyses

The nutrient concentration, moisture content, prokaryote abundance and enzymatic activity in all treatments were analyzed using one-way ANOVA. T-tests were used to determine whether the sediment moisture, nutrient concentration, prokaryote abundance and enzymatic activity differed over time. The analyses were performed using SigmaStat software for Windows version 2.03. Fisher’s exact test was used to determine the significance of the differences between pairs of probability values for multiple enzymatic functions. Differences in the enzyme activity patterns and the relationship of these differences to the total abundance of bacteria and the abundance of the bacterial taxa quantified through CARD-FISH were analyzed using the Principle Component Analysis (PCA), with enzyme activity patterns as active variables and the bacterial community data as supplementary variables, mapped into the factor structure determined from the active variables (Statistica 10.0 software package, Tulsa, OK).

## Results

### Desiccation Experiment

**Figure 1:**
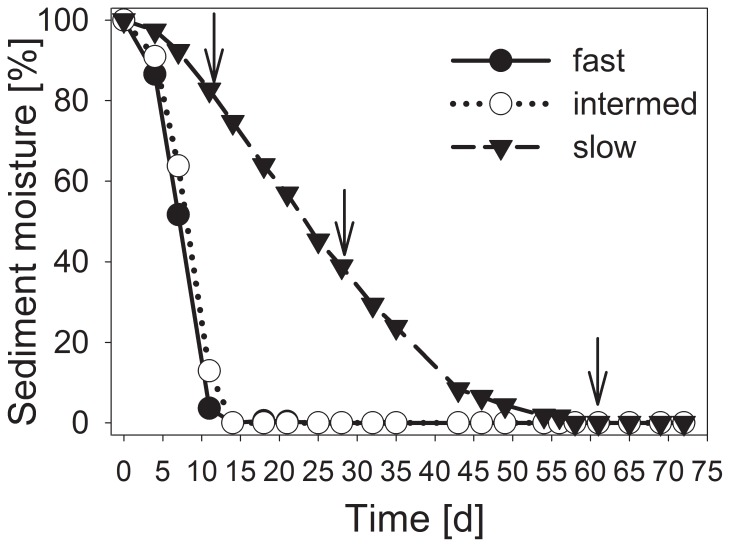
Water loss over time during the sediment desiccation process. Desiccation occurred at 20°C in polypropylene containers without cover (fast), with gaze (intermediate) or with plastic foil cover (slow desiccation scenario). The final moisture content of the sediment was set to 0%. The arrows indicate the sampling times (after 2, 4 and 8 weeks of desiccation).

Sediment moisture achieved a final level of desiccation (0% moisture) with the fast and intermediate desiccation treatments within 14 days (Fig. 1). In the slow drought trials, the sediment moisture decreased continuously until day 54. All drying scenarios showed differences in sediment moisture content until day 11 (ANOVA, P<0.05). After 14 days, the sediment moisture in the slow drought scenario differed from the fast and intermediate drought scenario until day 35 (ANOVA, P<0.05).

Thus, the investigations after 2 weeks were performed when the sediments from the fast and intermediate desiccation scenarios had reached the final water content. When performing the 8-week sampling, the slowly desiccated sediment achieved this level. The sediments from all treatments remained at this moisture condition until the final sampling 5 weeks later.

The C:N ratio did not significantly change in all 3 desiccation scenarios until the end of the experiment (Tables 1 and S2). The concentrations of nitrogen and nitrate increased in all treatments (ANOVA, P<0.05) but the increase was most pronounced in the slow desiccation scenario. The concentration of ammonia decreased in all treatments until the 4-week step (ANOVA, P<0.05), but at the end of the experiment, the difference was not significant compared with the initial concentration (Tables 1 and S2).

**Table 1:** Sediment chemical characteristics.

	weeks	fast	intermediate	slow
C:N ratio	0	8.8±0.8		
	2	8.7±1.2	9.4±1.3	9.6±1.3
	4	8.8±0.5	8.9±0.9	9.8±1.0
	8	9.0±0.5	9.1±0.8	11.1±2.7
	13	8.8±0.8	8.7±0.4	9.0±1.3
Nitrogen (µg N g dw^−1^)	0	1.61±0.22		
	2	2.46±0.29	2.78±0.48	7.19±3.46*
	4	2.00±0.82	2.93±0.33	5.14±0.84*
	8	2.46±0.27	2.65±0.15	6.13±0.15*
	13	2.68±0.21	2.73±0.28	5.93±1.76*
Nitrate (µg N g dw^−1^)	0	1.17±0.11		
	2	2.18±0.28	2.42±0.43	6.57±3.31
	4	1.77±0.88	2.64±0.34	4.82±0.17*
	8	2.13±0.32	2.36±0.21	5.88±0.33*
	13	2.18±0.13	2.35±0.24	5.59±1.92*
Ammonium (µg N g dw^−1^)	0	0.34±0.21		
	2	0.15±0.07	0.23±0.13	0.24±0.16
	4	0.12±0.05	0.15±0.04	0.14±0.02
	8	0.18±0.04	0.21±0.11	0.14±0.06
	13	0.41±0.10	0.30±0.11	0.24±0.24

C:N ratio, concentrations of nitrogen, nitrate, and ammonium in streambed sediments during different desiccation scenarios (fast, intermediate and slow). Means with SD are given (n = 4–5). The asterisks indicate significant differences between the treatments (ANOVA, * = P<0.05).

#### Bacterial community structure

The abundance of prokaryotes was decreased in all treatments after 2 weeks (ANOVA, P<0.05) and subsequently increased until week 13 (Tables 2, S3 and S4), which was significant between the 2- and 4-week steps (ANOVA, P<0.05) in all treatments. The increase was also significant with the intermediate treatment (ANOVA, P<0.05) between weeks 4 and 8.

**Table 2:** Abundance of prokaryotes in experimentally desiccated Breitenbach streambed sediments.

	weeks	fast	intermediate	slow
Prokaryotes	0	3.8±1.2		
(10^9^ cells mL^−1^)	2	2.5±1.0*	2.7±1.0*	2.3±0.8*
	4	5.0±1.1	4.4±0.8	4.5±1.3
	8	5.5±2.3	6.4±2.0	4.7±1.5
	13	4.1±2.3	6.1±1.4	5.0±2.4
*Bacteria*	0	1.3±0.3		
(10^9^ cells mL^−1^)	2	1.5±0.6	1.1±0.9	2.0±0.4
	4	1.0±0.3	0.6±0.3	1.5±0.6
	8	1.5±0.0	1.7±0.5	2.1±0.8
	13	1.7±0.2	2.1±0.6	1.4±0.3
*Alphaproteobacteria*	0	6.7±7.8		
(10^7^ cells mL^−1^)	2	29.4±13.0*	24.3±12.2*	30.6±22.4
	4	18.9±6.5	19.0±24.7	38.1±38.1*
	8	33.3±12.6*	31.1±35.0	23.0±6.1
	13	18.6±13.6	35.2±12.8*	23.3±14.8*
*Betaproteobacteria*	0	32.9±19.0		
(10^7^ cells mL^−1^)	2	12.0±0.7	15.6±3.1	23.8±14.4
	4	7.7±2.4*	8.5±4.4*	23.2±7.5
	8	12.8±9.7	11.0±4.8	13.5±8.9
	13	17.5±2.2	13.9±7.1	10.1±9.2
*Gammaproteobacteria*	0	3.4±2.6		
(10^7^ cells mL^−1^)	2	4.1±3.8	3.2±4.7	2.5±1.8
	4	4.9±3.8	3.0±2.3	5.3±4.4
	8	3.8±2.2	3.5±3.8	2.6±2.6
	13	1.0±1.7	0.7±0.9	6.3±6.8
*Bacteroidetes*	0	8.9±5.2		
(10^7^ cells mL^−1^)	2	4.8±3.2	3.4±1.7	3.9±4.1
	4	1.0±0.6	4.2±5.4	5.7±1.7
	8	1.4±0.8*	2.6±2.2	2.0±1.7
	13	1.9±0.5	3.8±2.4	2.2±2.4
*Actinobacteria*	0	3.6±2.2		
(10^7^ cells mL^−1^)	2	13.4±13.8	8.5±3.5	4.0±2.2
	4	8.9±3.5*	7.2±2.8	8.0±2.7*
	8	14.9±6.1*	21.1±3.5*	41.6±8.6**
	13	18.1±7.0*	17.1±13.1	21.3±13.8*
*Firmicutes*	0	4.3±4.1		
(10^7^ cells mL^−1^)	2	0.9±0.8	1.6±1.2	1.6±1.4
	4	4.9±3.1	2.9±3.1	1.2±1.3
	8	1.5±1.1	3.5±3.6	3.2±4.8
	13	3.9±2.1	1.7±1.3	1.7±1.3

The abundance of prokaryotes was determined after SYBR Green staining, whereas the abundances of different taxonomic groups were determined via CARD-FISH. Means with SD are given (n = 4). The asterisks indicate significant differences between wet sediment from day 0 and the treatment samples (ANOVA, * = P<0.05, ** = P<0.01).

**Figure 2:**
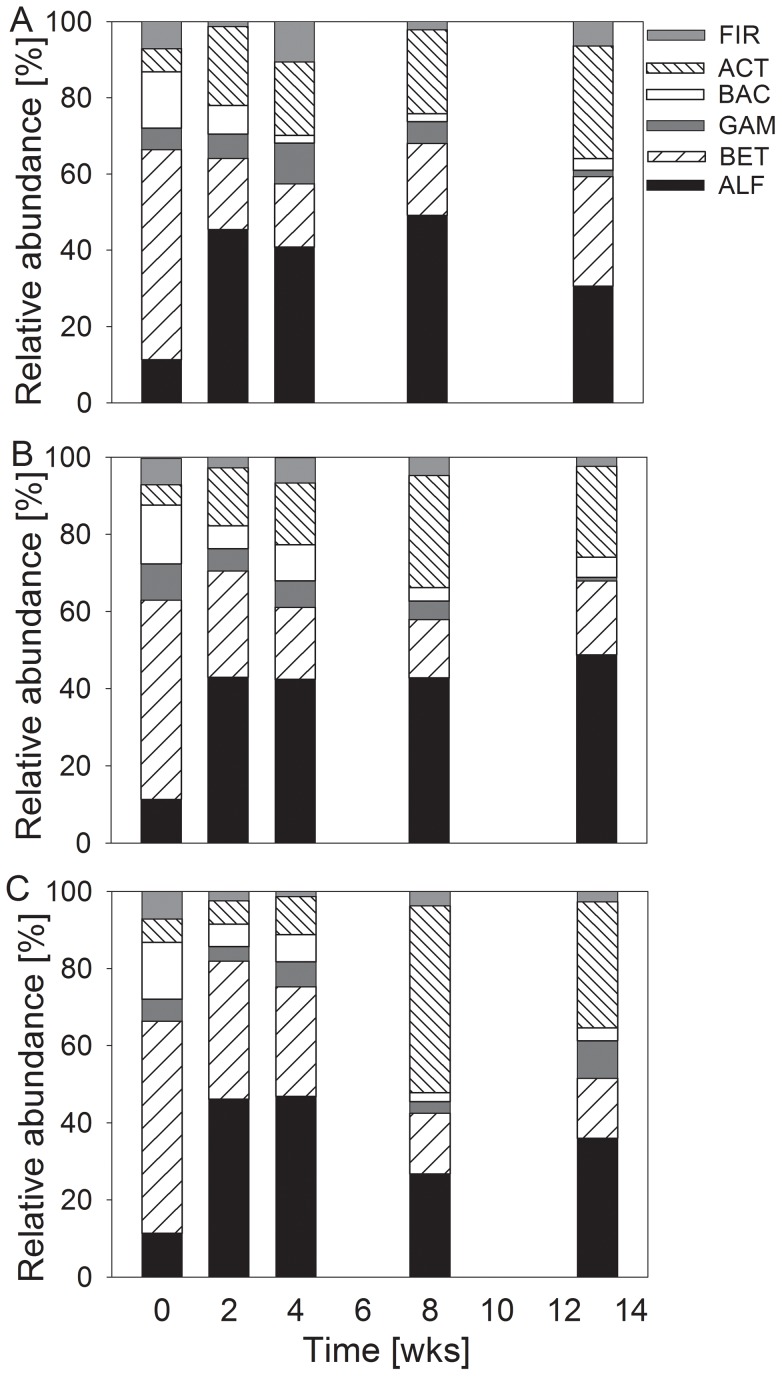
Development of bacterial community composition in desiccating Breitenbach sediment. The relative abundances of the different taxonomic groups are given for wet sediment (week 0) and after 2, 4, 8 and 13 weeks of different artificial desiccation: fast (A), intermediate (B) and slow desiccation (C). The determined groups were *Alphaproteobacteria* (ALF), *Betaproteobacteria* (BET), *Gammaproteobacteria* (GAM), *Bacteroidetes* (BAC), *Actinobacteria* (ACT) and *Firmicutes* (FIR) (n = 4).

The initial bacterial community in the wet streambed sediment primarily contained *Proteobacteria*, achieving approximately 70% of all affiliated cells (*Beta*- 55%, *Alpha*- 11%, and *Gammaproteobacteria* 5.6%; Fig. 2). The phyla of *Bacteroidetes* (15%), *Actinobacteria* (6.1%) and *Firmicutes* (7.2%) were much less abundant. During desiccation, the bacterial community changed in all treatments towards higher proportions of *Alphaproteobacteria* and *Actinobacteria* and lower proportions of *Betaproteobacteria* and *Bacteroidetes* (Fig. 2 A–C, Tables 2, S3 and S4).

The total number of bands of identical position in all samples, identified in the TGGE pattern characterizing bacterial community composition, was 30, deviating between 14 and 23 bands per sample. No trend could be identified for diversity (Shannon-Wiener diversity H_s_ ranging from 2.42 to 3.02) and evenness (J, ranging from 0.86 to 0.98) during desiccation or between the scenarios.

**Figure 3:**
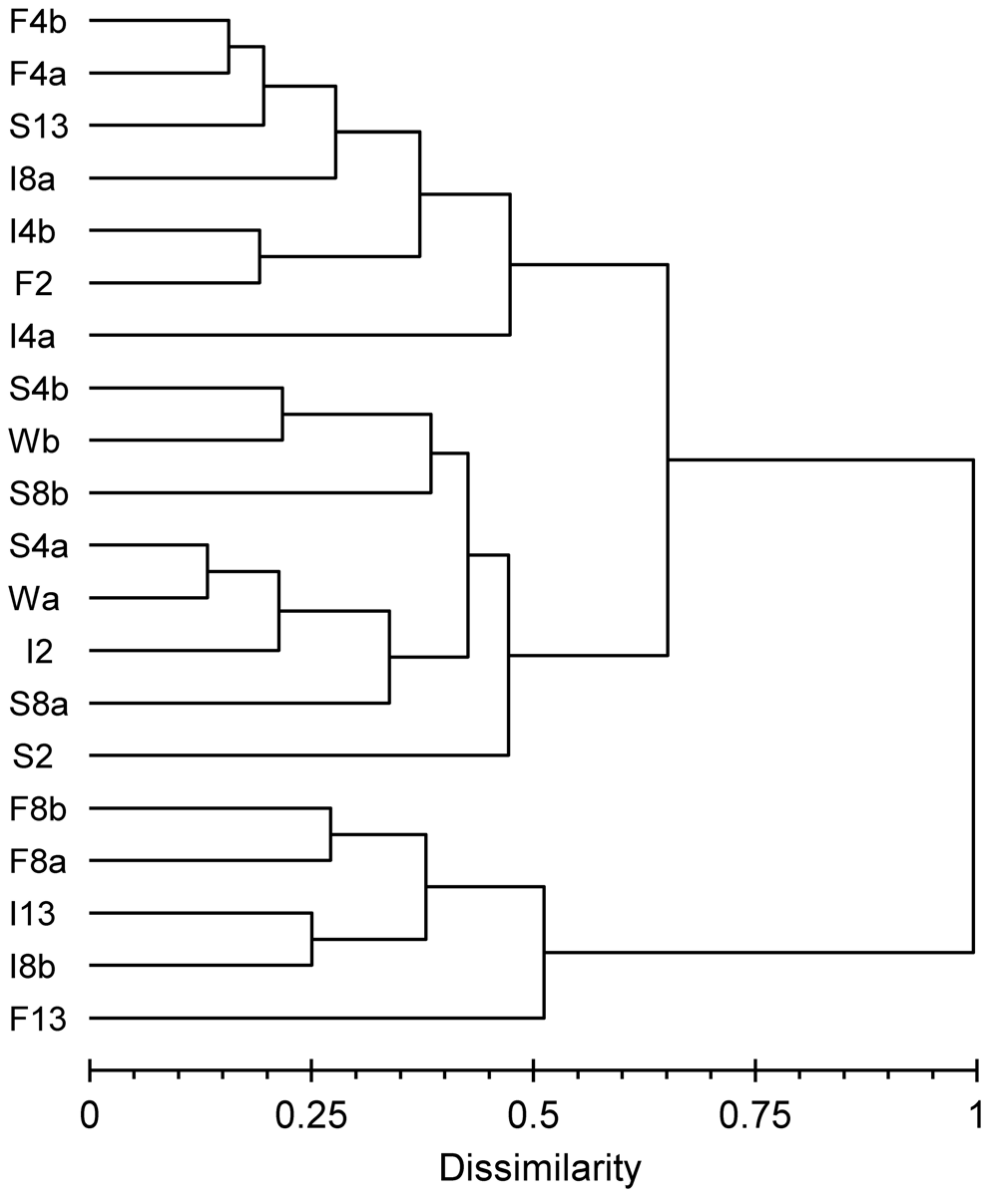
Comparison of the bacterial community composition in desiccating Breitenbach sediment via cluster analysis. Cluster analysis was performed with TGGE profiles prepared with 16S rRNA gene fragments. Bray-Curtis dissimilarities were calculated for each pair of lanes; the dendrogram was constructed using the Ward method. W = initial wet samples, F = fast desiccation, I = intermediate desiccation, S = slow desiccation. The numbers indicate the weeks of desiccation, and the small letters denote the replicates.

The bacterial communities from the different samples analyzed by cluster analyses using TGGE profiles were separated in the first step into two main groups (Fig. 3) containing the samples from fast and intermediate desiccation scenarios after 8 and 13 weeks (F8, F13, I8, and I13), except one (I8a) of the 8-week intermediate samples. The second major cluster was divided into two subclusters. One subcluster contained the initial wet sediment (W), the samples from the slow desiccation treatment until 8 weeks of desiccation (S2, S4, and S8) and the sediment after 2 weeks of intermediate treatment (I2). The other subcluster contained the remaining samples, which included the 2- and 4-week samples from the fast drought scenario (F2 and F4), both 4-week samples and one of the 8-week sample from the intermediate scenario (I4 and I8a) and the sediment desiccated slowly over 13 weeks (S13).

**Figure 4:**
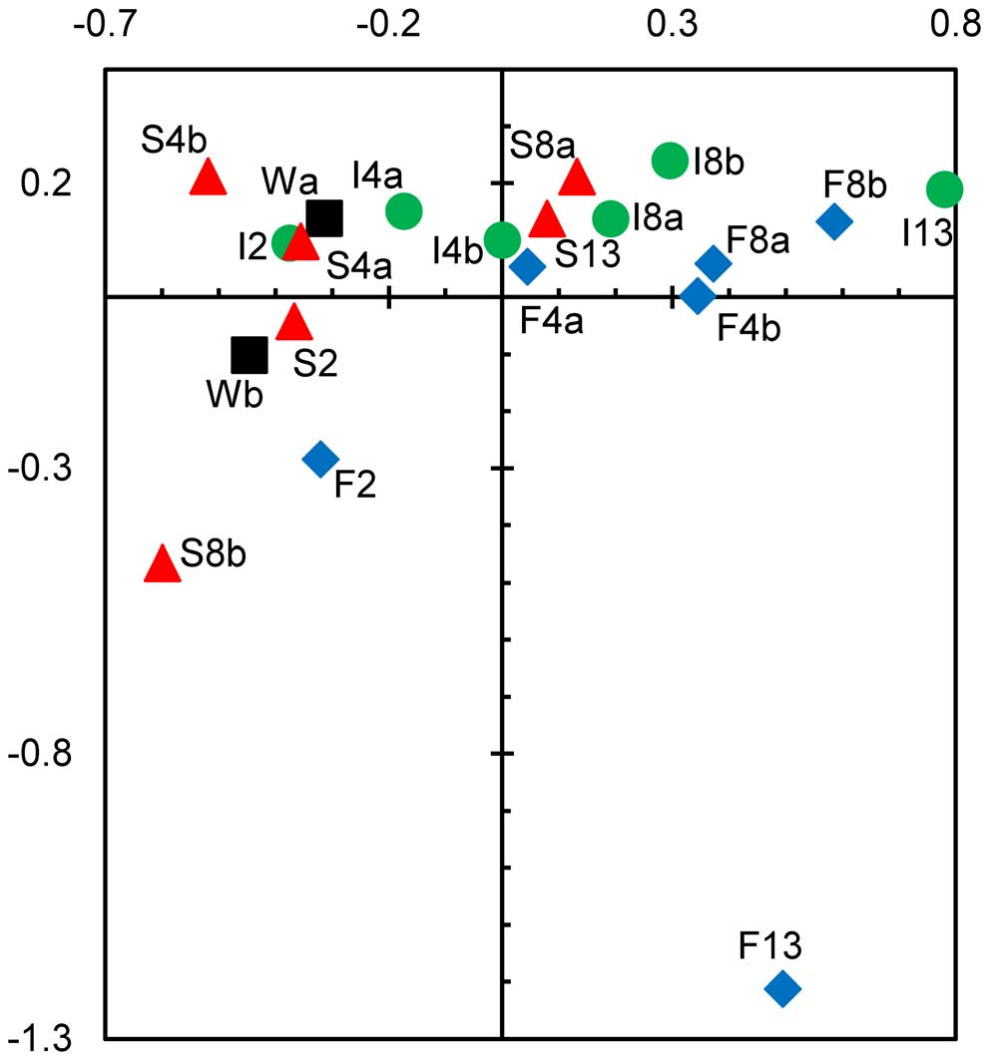
Comparison of bacterial community composition in desiccating Breitenbach streambed sediment via correspondence analysis. The analysis is based on TGGE band patterns prepared with 16S rRNA gene fragments. Axes 1 and 2 explain 23 and 17% of the variance in bacterial community composition, respectively. The black boxes symbolize initial wet samples (W), the blue diamonds indicate fast desiccation (F), the green circles indicate intermediate desiccation (I) and the red triangles indicate slow desiccation (S). The numbers indicate the weeks of desiccation, and the small letters denote the replicates.

TGGE profiles are summarized in a correspondence analysis plot (Fig. 4), confirming the major trends from the cluster analysis. Axes 1 and 2 explained 40% of the bacterial community variation. The wet samples (W) were grouped together with the samples after 2 weeks of desiccation from all treatments (F2, I2, and S2) and the samples from the slow desiccation treatment after 4 weeks (S4), indicating similar community composition. With ongoing desiccation, the community composition becomes increasingly different from the composition of the original streambed community. This shift was much more pronounced with the fast and intermediate desiccation scenarios (F4, F8, F13, I4, I8 and I13), whereas the composition of the bacterial community in the slow desiccation process after 13 weeks (S13) was much closer to the initial community composition, similar to the structure observed after 4 weeks of fast and intermediate desiccation treatments (F4 and I4) and 8 weeks of intermediate desiccation (I8).

#### Extracellular enzyme activity

**Figure 5:**
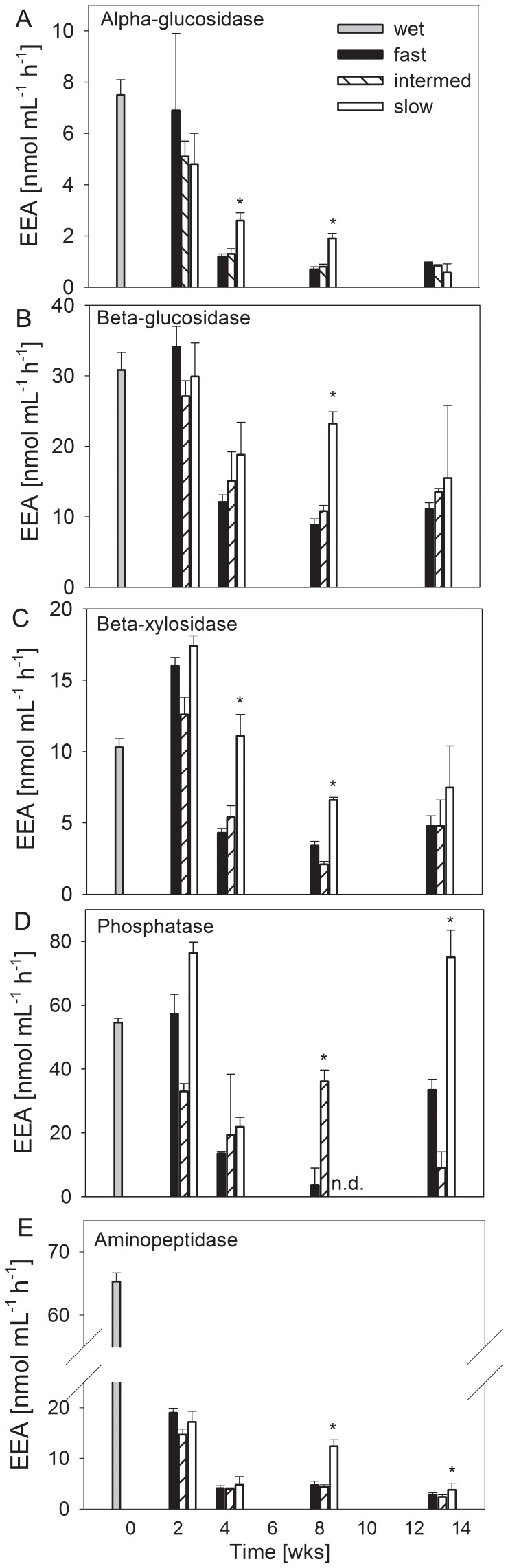
Activities of extracellular enzymes in desiccating Breitenbach sediment. The activities were determined in wet sediments (0) and after 2, 4, 8 and 13 weeks of artificial desiccation, using 3 different scenarios: fast, intermediate and slow desiccation. (A) alpha-glucosidase, (B) beta-glucosidase, (C) beta-xylosidase, (D) phosphatase, (E) aminopeptidase. Mean values with SD are given (n = 4). The asterisks indicate significant differences between the treatments (ANOVA, P<0.05).

The responses of the activities of the measured enzymes were different with respect to the treatments (Fig. 5, Table S5). In general, the activity of aminopeptidase was most affected, decreasing to approximately 10% of the initial values in all treatments after 2 weeks (90% reduction) and to less than 10% after 13 weeks of desiccation (Fig. 5E). Alpha-glucosidase was also distinctly affected, decreasing to 13% after 13 weeks, and phosphatase was decreased to 40% of the initial values (60% reduction) after 4 weeks in all treatments (Fig. 5A, D). Beta-xylosidase activity exhibited a less pronounced decrease to 50% of the initial values with the fast and intermediate treatments after 4 weeks and all treatments after 13 weeks of desiccation (Fig. 5C). Beta-glucosidase activity remained at approximately 50% of its initial value in all treatments, even after 13 weeks. In the slow drought scenario, the activities were higher than in the other treatments (ANOVA, P<0.05) for alpha-glucosidase and beta-xylosidase after 4 weeks, for alpha- and beta-glucosidase, beta-xylosidase and aminopeptidase after 8 weeks and for phosphatase and aminopeptidase after 13 weeks. All extracellular enzymes under investigation were identified through activity measurements as persistent until 13 weeks of desiccation, although the lowest reduction in activity was detected with the slow drought treatment (Fig. 5).

**Figure 6:**
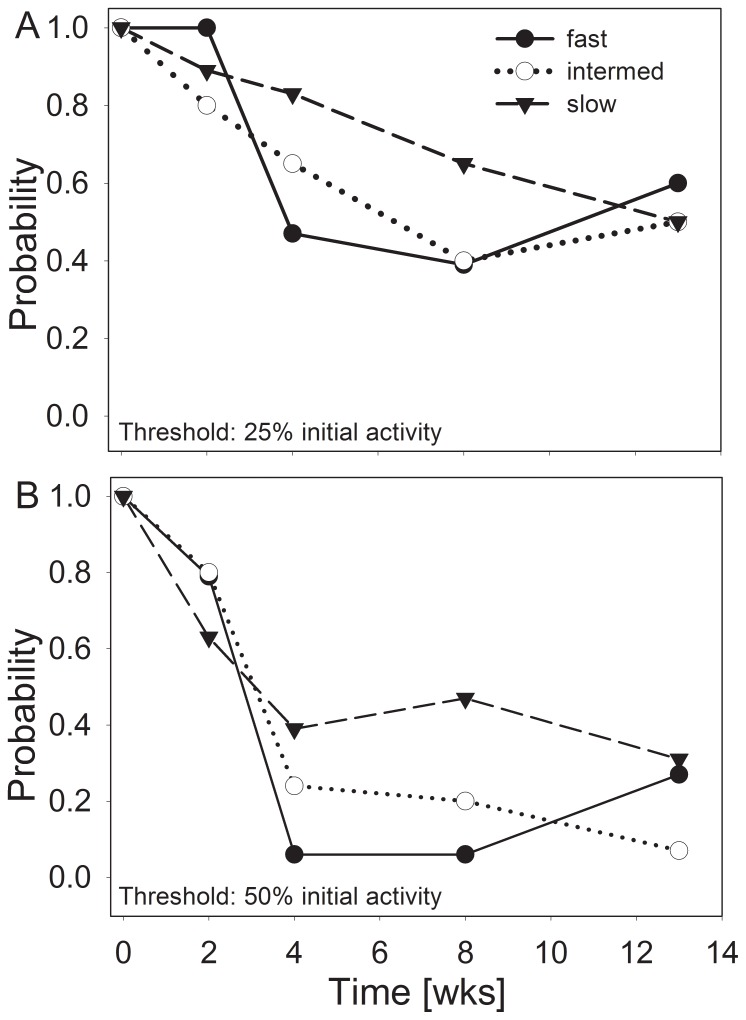
Probabilities for sustaining extracellular enzymatic functions during the process of sediment desiccation. Threshold levels of 50% and 25% of the initial activity in unaffected sediments are used. Probabilities were calculated from single measurements for 5 enzymes (n = 14–20).

The probabilities for sustaining multiple extracellular enzymatic functions decreased during the desiccation process with respect to both threshold levels used (Fig. 6). With the 25% threshold, the multiple enzymatic activity was reduced with the intermediate (P<0.001) and fast desiccation scenarios after 4 weeks (P<0.05), whereas with the slow treatment, the decrease was gradual and not significant until 8 weeks. The decrease was particularly pronounced with the fast treatment between the 2- and 4-week steps (P<0.001). With the 50% threshold, the probability values were significantly decreased with the slow treatment after 2 weeks (P<0.01). However, the loss of enzymatic community functions was more pronounced (P<0.001) with the intermediate and fast treatments than with the slow desiccation treatment after 4 weeks. At the end of the desiccation experiment, no significant differences were detected between the 3 treatments (Fig. 6).

**Figure 7:**
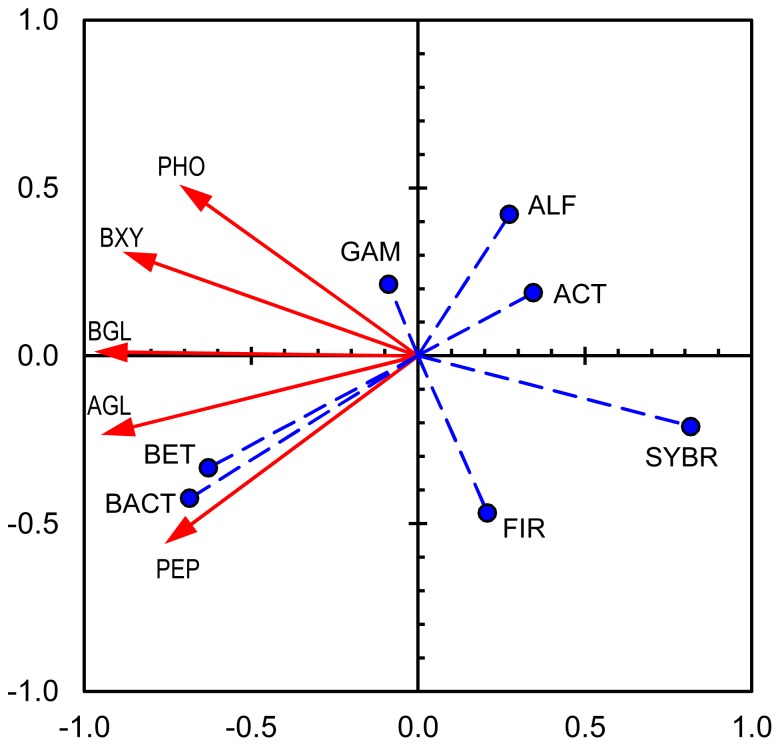
PCA biplot of extracellular enzyme activities and abundance of bacteria in desiccating Breitenbach sediment. Axes 1 and 2 explain 75% and 15% of the variance in enzyme activity, respectively. The red arrows with continuous lines represent extracellular enzyme activities (active variables; AGL = alpha-glucosidase, BGL = beta-glucosidase, BXY = beta-xylosidase, PHO = phosphatase, PEP = aminopeptidase); the blue circles with dashed lines symbolize the bacterial abundances (supplementary variables; SYBR = prokaryotes determined via SYBR green staining, ALF = *Alphaproteobacteria*, BET = *Betaproteobacteria*, GAM = *Gammaproteobacteria*, BACT = *Bacteroidetes*, ACT = *Actinobacteria*, FIR = *Firmicutes*).

The activities of extracellular enzymes and their relationships with the bacterial community structure were summarized using a PCA biplot (Fig. 7). Axis 1 explained 75% of the variance (eigenvalue 3.7). Axis 2 explained only 15% of the variance (eigenvalue 0.8). *Betaproteobacteria* and *Bacteroidetes* showed close relationships, particularly with aminopeptidase, whereas *Alphaproteobacteria* and especially *Actinobacteria* exhibited contrasting loadings, particularly when axis 1 is considered. In addition, the total number of bacteria developed contrary to the enzyme activities. *Gammaproteobacteria* and *Firmicutes* showed no distinct relationships with enzyme activities, exhibiting small loadings at least on axis 1.

### Rewetting Experiment

#### Bacterial community structure

In the treatment with unfiltered stream water (containing bacterial cells), after 10 and 14 days of exposition, prokaryotes achieved higher abundances (1.8 to 2.6-fold) than those observed in the dry sediment, showing abundances even higher than those observed in the initial wet sediment before desiccation (ANOVA, P<0.05; Table 3, Table S6).

**Table 3:** Abundances of prokaryotes in Breitenbach streambed sediments experimentally rewetted after 13 weeks of desiccation.

treatment	days	Prokaryotes	*Bacteria*	*Alphaproteobacteria*	*Betaproteobacteria*	*Gammaproteobacteria*	*Bacteroidetes*	*Actinobacteria*	*Firmicutes*
		(10^9^ cells mL^−1^)	(10^9^ cells mL^−1^)	(10^7^ cells mL^−1^)	(10^7^ cells mL^−1^)	(10^7^ cells mL^−1^)	(10^7^ cells mL^−1^)	(10^7^ cells mL^−1^)	(10^7^ cells mL^−1^)
dry	0	4.1±2.3	1.7±0.2	18.6±13.6	17.5±2.2	1.0±1.7	1.9±0.5	18.1±7.0	3.9±2.1
with cells	1	3.9±1.2	1.2±9.7	33.1±13.5	50.2±19.2*	16.2±4.3*	21.8±11.2	20.0±16.5	11.1±17.9
	2	3.1±1.8	1.0±0.3	44.2±22.6	111.7±2.8**	17.3±14.5	18.7±20.5	5.2±3.7*	5.2±4.2
	3	3.6±0.9	1.9±2.1	49.7±24.9	128.7±102.2*	13.8±9.4*	42.4±30.0*	8.3±5.7	3.5±2.0
	6	5.4±2.6	2.0±2.7	32.3±17.5	235.0±141.8	25.0±17.3	11.8±9.9	11.2±11.2	5.3±3.3
	10	10.7±5.6*	2.7±2.8	107.2±18.5*	115.6±133.8	8.9±8.7	16.8±7.3*	12.8±4.7	8.9±6.7
	14	7.5±0.9*	2.6±1.1	100.7±73.1	128.2±65.0*	14.7±14.8	10.0±5.0	5.2±3.0	10.6±3.6*
without cells	1	2.8±1.8	1.6±0.7	30.4±12.9	33.6±7.6*	8.3±6.7	11.0±7.3	4.3±4.4	8.0±6.3*
	2	2.1±1.3	0.5±0.3	50.9±36.2	46.4±23.1	14.6±13.9	4.3±4.4	14.7±20.8	10.4±3.0*
	3	3.6±2.1	1.3±9.6	59.4±59.3	190.5±111.2	67.2±23.3*	11.1±11.6	7.9±1.3	13.1±4.7
	6	2.8±1.5	1.1±1.7	17.6±15.9	53.3±41.7	33.1±52.8	26.1±16.4*	15.1±13.2	9.6±7.4
	10	6.4±1.8	0.8±0.7	28.0±21.2	45.1±13.0*	47.8±41.9	12.0±7.3	5.3±2.6*	5.7±4.0
	14	4.2±1.7	0.9±0.5	37.3±25.4	74.0±25.4*	7.4±2.2*	8.4±1.5*	6.7±1.1*	7.4±1.1

The abundance of prokaryotes was determined after SYBR Green staining, whereas the abundances of different taxonomic groups were determined via CARD-FISH. Means with SD are given (n = 4). The asterisks indicate significant differences between dry sediment from day 0 used for rewetting and the treatment samples (ANOVA, * = P<0.05, ** = P<0.01).

**Figure 8:**
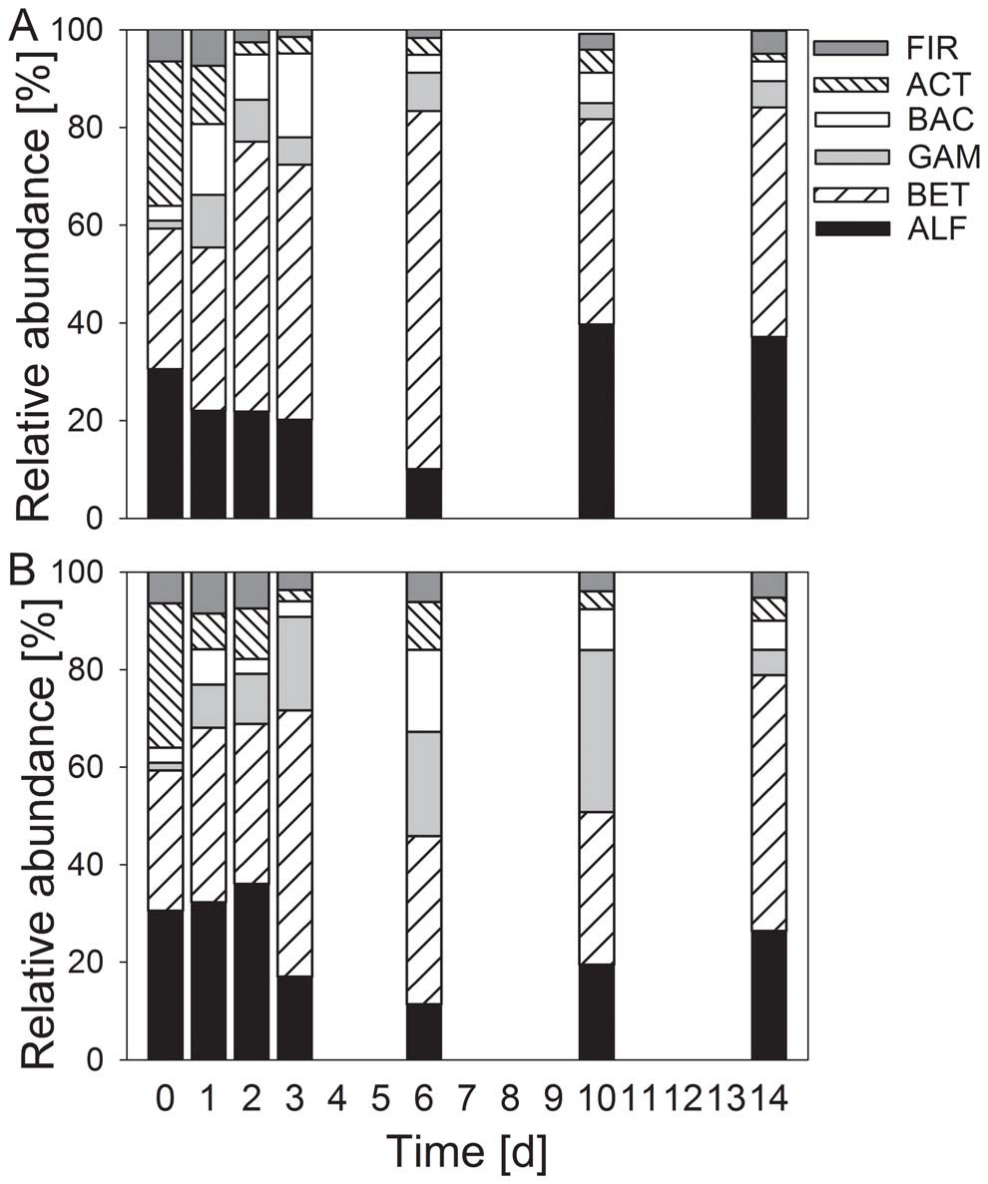
Development of bacterial community composition in desiccated Breitenbach sediment during rewetting. The relative abundances of different taxonomic groups are given upon rewetting (A) with untreated stream water containing the natural community of microorganisms and (B) with sterile (filtered and boiled) stream water. Means (n = 4) before and after 1, 2, 3, 6, 10 and 14 days of rewetting are given. For abbreviations see Fig. 2.

During the rewetting process, the bacterial community composition changed again but did not obtain the pre-desiccation composition (Fig. 8). After 14 days of rewetting, higher proportions of *Alphaproteobacteria* and lower proportions of *Bacteroidetes* persisted compared with the initial wet streambed sediment.

The abundance of nearly all investigated groups increased during rewetting (Table 3), with the exception of *Actinobacteria* which declined (ANOVA, P<0.05). After 14 days of rewetting, the abundances of *Alphaproteobacteria* and *Actinobacteria* were higher with the rewetting approach using water with cells compared with water without cells (ANOVA, P<0.05) (Fig. 8, Table 3).

The TGGE gel prepared for analyzing the development of the bacterial community composition during rewetting of the desiccated sediment facilitated the identification of 38 bands of identical position in total, with deviations between 10 and 18 bands per sample. Moreover, no trend could be identified for diversity (Shannon-Wiener diversity H_s_ ranging from 1.87 to 2.72) and evenness (J, ranging from 0.73 to 0.94) during the two-week recovery process or between the different types of perfusion water.

**Figure 9:**
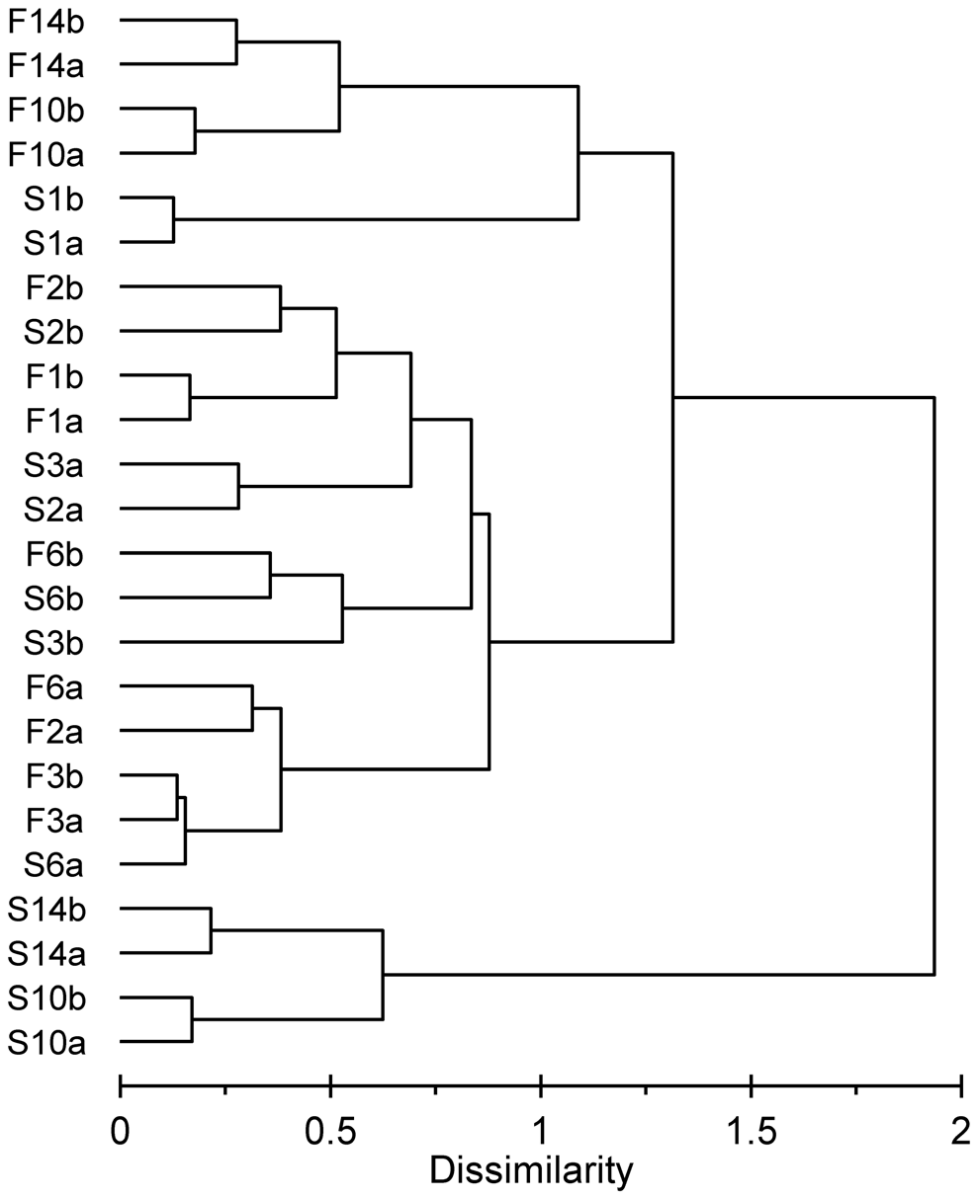
Comparison of bacterial community composition in rewetted Breitenbach sediment after artificial desiccation via cluster analysis. The dendrogram was prepared as described in Fig. 4. S = sediment perfused with unaffected Breitenbach stream water containing the natural microbial community, F = sediments perfused with filtered and boiled stream water containing no microorganisms. The numbers indicate the days of rewetting and small letters indicate the replicates.

The cluster analysis (Fig. 9) showed the clear separation of the sediments perfused with untreated stream water after 10 and 14 days of perfusion (S10, S14) and the sediments perfused with sterile stream water after 10 and 14 days (F10, F14) from all other samples (up to 6 days of perfusion). No obvious grouping could be detected within the remaining communities, but the samples perfused with stream water for one day (S1) were grouped closer to the F10 and F14 samples.

**Figure 10:**
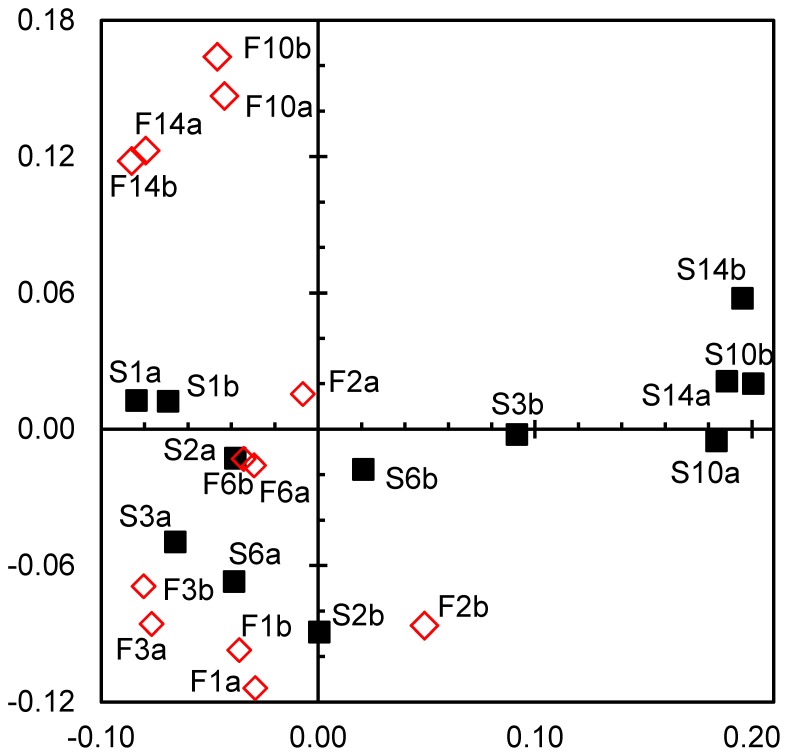
Comparison of bacterial community composition in rewetted Breitenbach sediment after artificial desiccation via correspondence analysis. The analysis is based on TGGE band patterns prepared with 16S rRNA gene fragments. Axes 1 and 2 explain 23 and 16% of the variance in bacterial community composition, respectively. The filled black boxes symbolize sediments perfused with unaffected Breitenbach stream water containing the natural microbial community (S), and the open red diamonds indicate sediments perfused with filtered and boiled stream water containing no microorganisms (F). The numbers indicate the days of rewetting, and the small letters denote the replicates.

The correspondence analysis (Fig. 10) exhibited similar trends for the bacterial community composition, characterized by the TGGE patterns. Axes 1 and 2 explained 39% of the community variation. Most samples from the first 6 days of rewetting are closely assembled (S1– S6 and F1– F6). However, during recovery, the composition of the bacterial communities changed, whereas distinct development was observed when the sediments were rewetted with the 2 different types of perfusion water (untreated stream water S10 and S14 or filtered sterile stream water F10 and F14).

#### Extracellular enzyme activity

**Figure 11:**
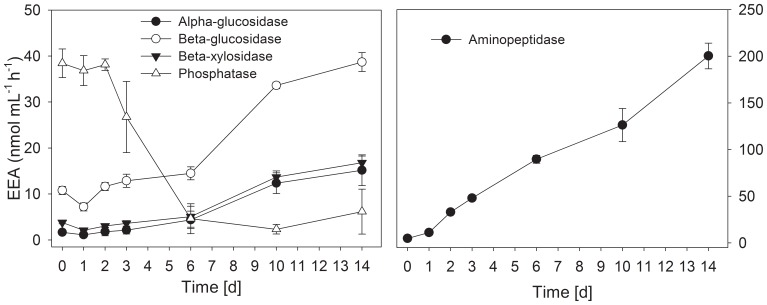
Activities of extracellular enzymes in rewetted Breitenbach sediment after artificial desiccation. Activities were determined at the onset of rewetting and after 1(n = 4). Detailed data are presented in Table S6.

The initial levels of extracellular enzyme activity measured, when initiating the rewetting process with sterilized stream water only, were similar to the values determined after 13 weeks of fast desiccation (Fig. 11, Table S7), although different measuring approaches were used (suspension technique vs. perfused core technique). Only alpha-glucosidase (0.94 nmol L^−1^ h^−1^ vs. 1.7 nmol L^−1^ h^−1^) and aminopeptidase (2.8 nmol L^−1^ h^−1^ vs. 6.9 nmol L^−1^ h^−1^) showed comparatively large deviations, whereas deviations below 20% were observed for the other enzymes. With the exception of phosphatase, all enzyme activities increased over time. This increase was particularly rapid for aminopeptidase, showing a significant increase (compared with the initial values) after 3 days; the increase was not significant for alpha- and beta-glucosidase until 10 days of rewetting (t-test, P<0.05). However, phosphatase exhibited less activity after 6 to 14 days of rewetting compared with the initial activity (t-test, P<0.05; Fig. 11, Tables S7).

**Figure 12:**
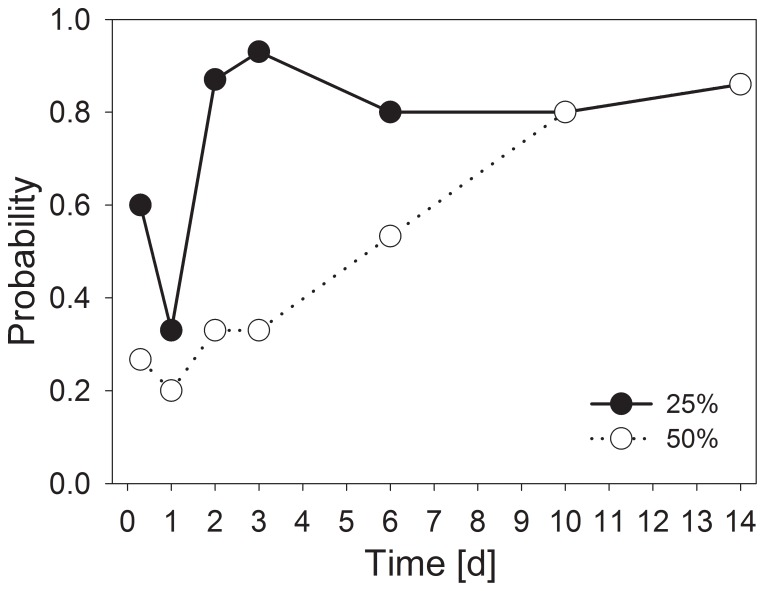
Probabilities for sustaining extracellular enzymatic functions during the process of rewetting artificially desiccated sediment. Threshold levels of 50% and 25% of the initial activity in unaffected sediments are used. The probabilities were calculated from single measurements for 5 enzymes (n = 15).

The probabilities for sustaining multiple extracellular enzymatic functions remained at levels similar to the desiccated sediment throughout the first hours of rewetting (Fig. 12) for both threshold values (25% and 50%). With the 25% threshold, a short decrease appeared after 24 h (P<0.05), reflecting decreased phosphatase activity. However, multiple enzymatic functions nearly recovered completely after 48 h, maintaining at least 0.8 probability until the end of the experiment after 2 weeks. With the 50% threshold value, this level had been achieved later, showing significance (P<0.001) at 10 days after the initiation of rewetting (Fig. 12). The total recovery of enzymatic community functionality was not achieved because phosphatase activity decreased a few hours after rewetting (Fig. 12, Tables S7).

**Figure 13:**
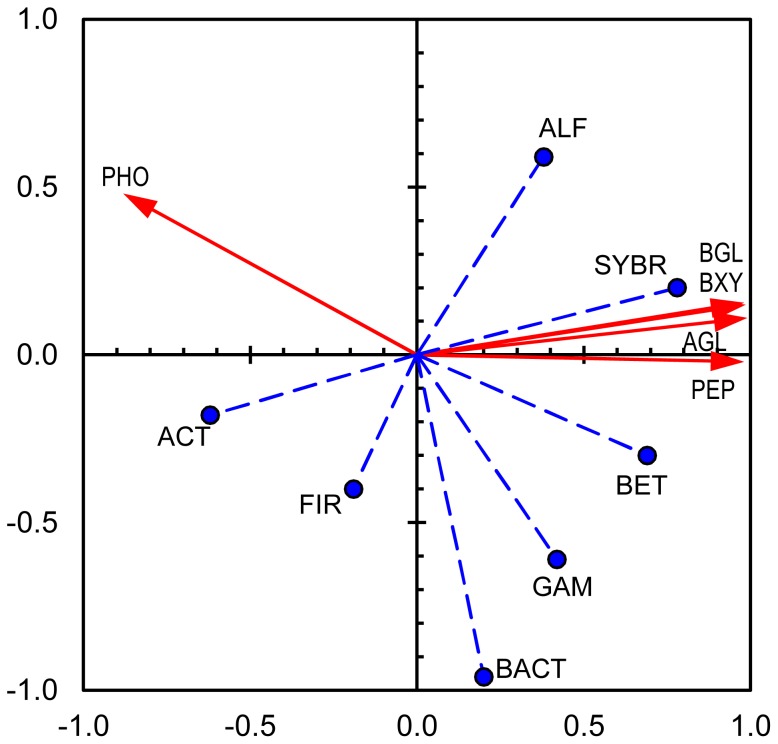
PCA biplot of extracellular enzyme activities and bacterial abundance in rewetted Breitenbach sediment after desiccation. Axes 1 and 2 explain 92% and 5% of the variance in enzyme activity, respectively. For further explanations cf. Fig. 7.

The PCA biplot of EEAs (active variables) and the bacterial community structure (supplementary variables) confirmed the differences in EEA development during rewetting described for phosphatases compared with all other enzymes (Fig. 13). Axis 1 explains 93% of the variance for the enzyme data (eigenvalue 4.7), whereas axis 2 explained only 6% (eigenvalue 0.3) of the variance. Considerable relationships with EEAs (except with phosphatase activity) can be observed for the total number of bacteria and to a lesser degree for *Betaproteobacteria*, with an opposite relationship observed for *Actinobacteria*. All other bacterial groups exhibited low loadings on axis 1 (*Alpha-*, *Gammaproteobacteria*, *Bacteroidetes*, *Firmicutes*) but mostly high loadings on the less important axis 2 (*Alpha-*, *Gammaproteobacteria*, *Bacteroidetes*).

## Discussion

### Desiccation

The rapid decrease of moisture was similar to microcosm experiments with Mediterranean stream sediment desiccated at 18°C [35]. In temperate Central European streams, desiccation occurs slower and less drastically than in Mediterranean streams, although fast and intense desiccation has already occurred under specific local conditions and occurs more frequently [37], [53]. However, the slow desiccation scenario likely simulates most of the desiccation events occurring in Central Europe.

Variations in hydrology have strong effects on nutrient cycling processes in stream ecosystems [34], [54]. In the present study, the total nitrogen and nitrate increased during desiccation, consistent with earlier studies (2004) on Breitenbach sediments [7], where higher total N concentrations were observed in naturally desiccated sediment (20 µg N g dw^−1^) than in wet sediment (4 µg N g dw^−1^) (Marxsen & Zoppini pers. comm.). Similar to the Breitenbach study (Table 1), in the present study, the concentration of ammonium declined until the end of the experiment at 18 days, reflecting the stimulation of N-mineralization and nitrification [35].

The community composition of stream bacteria is associated with the carbon source available [55]. The C:N ratio in aquatic environments increases with high proportions of detritus (30) and primary producers (20) and decreases with high proportions of consumers (10) [56], suggesting a high proportion of consumers in the stream sampled in the present study (Table 1).

The total number of prokaryotes (3.8×10^9^ cells mL^−1^; Table 2) in the wet sediment sampled from the Breitenbach was within the range typical for this stream [57]. In addition, the composition of the community determined via CARD-FISH was similar to that observed in previous studies [29], [30], [39], showing predominantly *Proteobacteria* and smaller amounts of *Bacteroidetes* and the Gram-positive phyla *Firmicutes* and *Actinobacteria* (Fig. 2, Table 2). Consistent with studies from Mediterranean streams [5], the abundance of prokaryotes in desiccating Breitenbach sediments was decreased in all treatments after 2 weeks. After 4 weeks of artificial drought, the abundances recovered, accompanied by the development of higher abundances of *Alphaproteobacteria*, *Actinobacteria* and *Firmicutes* (Fig. 2, Table 2). This trend was more distinct with the intermediate and slow drought scenarios. Gram-positive bacteria are more resistant to low water content [23], [31] and thus have an advantage over Gram-negative bacteria during drought events. These three groups are usually predominant in soil communities [58], suggesting that the bacterial community composition tended towards the development of soil community structures after this short time interval of desiccation. *Alphaproteobacteria* are predominantly found in C-poor stream and soil ecosystems [55], [58], suggesting that environmental conditions in desiccating stream sediments also change with the low availability of carbon.

The CARD-FISH approach used in the present study for the analysis of bacterial community composition only detects changes between larger taxonomic groups. However, the TGGE analysis compares the development of community composition on the basis of OTUs used as surrogates for bacterial species. This fingerprint approach detects changes at the species level occurring at a DNA content of 0.1 to 1% [59], although this approach also presents several potential biases (e.g., [60], [61]). TGGE band patterns were evaluated via cluster and correspondence analyses. Both approaches confirmed the principal trends observed with the evaluation of the CARD-FISH data from the desiccation experiment. With increasing desiccation, bacterial communities became more and more different from the initial composition in wet sediments (Figs. 3 and 4). However, detailed analysis of TGGE band patterns demonstrated that the bacterial community did not change as fast in the slow desiccation scenario as in the more intense desiccated sediments of the other scenarios (Figs. 3 and 4).

Prokaryotes are important organisms for utilizing organic matter in small streams and transferring organic matter to higher trophic levels [12]. These organisms produce extracellular enzymes of special importance in the first step of macromolecular organic matter utilization and are of great importance in the food web and for nutrient regeneration [15]. Although extracellular enzymes are generally considered short-lived, a few studies have demonstrated that these molecules might persist during phases of dryness, e.g., in desiccated stream sediments [7], [37], aquatic biofilms [19], [62] and soil (e.g., [63]).

In the present study, we confirmed the general trend [7], [37] of decreasing potential extracellular enzyme activity in desiccating streambed sediments (Figs. 5 and 6). The evaluation of the results through the determination of multiple enzyme functions suggests that enzyme inactivation achieved a stable level after 4 to 8 weeks of desiccation and did not proceed until 13 weeks (Fig. 6). However, if desiccation occurs slowly, the process of enzyme inactivation occurs more slowly but continuously (Fig. 6).

Distinct differences between different enzymes are obvious. Aminopeptidases were most affected. These enzymes exhibited the fastest decrease and the lowest final activity (Fig. 5E). Enzymes involved in the degradation of polymeric carbohydrates (alpha-, beta-glucosidases, beta-xylosidases) were less affected. The activity of beta-glucosidases and beta-xylosidases slowly decreased below their initial activity potential in wet sediments (Fig. 5B, C), whereas the activity of alpha-glucosidase, which is involved in the decomposition of starch [30], decreased somewhat faster (Fig. 5A). No clear trend was detected for phosphatases, which exhibited irregular fluctuations. However, phosphatases have been shown to be more stable during drought periods in soil, as a previous study reported that 65% of indigenous enzyme activity was maintained after 7 weeks of soil storage at 22°C [63].

The analysis of the concordant developments between EEAs and bacterial community structure via CARD-FISH showed the closest relationships between aminopeptidase with *Betaproteobacteria* and *Bacteroidetes* (Fig. 7). Both taxa are not far from alpha- and beta-glucosidases in the biplot graphic and are also related to beta-xylosidase and phosphatase (axis 1 explains 75% of variance). In contrast, *Actinobacteria* exhibited inverse relationships with enzyme activity compared with these groups, which is also less distinctly true for *Alphaproteobacteria*. Thus, this analysis confirms the observation that during desiccation, the abundances of *Betaproteobacteria* and *Bacteroidetes* decreased, whereas those of *Actinobacteria* and *Alphaproteobacteria* increased, and the EEA decreased, particularly the aminopeptidase activity. However, no further relationship could be established between the abundances of bacterial groups, preferential enzyme activities and/or the velocity of drying. Surprisingly, the total number of bacteria exhibited an opposite development to EEA. However, the method used in the present study captures active and potentially active bacteria (including those from expanding taxa) and dormant and dead cells, whereas with the CARD-FISH approach only active and potentially active cells are covered [64].

### Rewetting

The number of bacteria in the desiccated sediment used for rewetting (4.1×10^9^ cells mL^−1^) was close to the initial value in the wet sediment (Tables 3 and S6). After rewetting, the number of bacteria fluctuated around this size. A significant increase in the number of prokaryotes to 10.7 and 7.5×10^9^ cells mL^−1^, respectively, was observed after 10 and 14 days of rewetting with cell-containing water. This suggests that cell-containing water enhances the development of the bacterial community. The composition of the bacterial community developed towards the initial structure but did not entirely recover to the structure observed in the aquatic sediments using both types of water (Fig. 8, Table 3); *Alphaproteobacteria* remained at predominant proportions and *Bacteroidetes* showed lower proportions even after 14 days of rewetting. During the experimental rewetting of desiccated sediment from a Mediterranean stream [32], the bacterial community structure remained dominated by *Alphaproteobacteria*. We observed a similar community structure, with *Alphaproteobacteria* representing 30% (rewetting with original stream water) and 25% (sterile stream water) of the community after 14 days of rewetting. During the first 3 days of recovery with water containing bacterial cells, *Betaproteobacteria* became more abundant, consistent with the high abundance of these bacteria in Breitenbach stream water [29]. However, the complete recovery of the bacterial community structure in desiccated temperate streambed sediments to a typical aquatic community likely requires more than 14 days of rewetting. Thus, after multiple desiccation events per year or after long dry seasons, the community might undergo a permanent change in the composition, resulting possibly in functional changes, too [7].

The analysis of the TGGE band patterns from the rewetting experiment showed somewhat different results compared with the CARD-FISH data evaluation. No clear trend in the community changes was observed within the first days of rewetting. However, the communities clearly deviated from the initial structure (at beginning of rewetting) after 10 and 14 days (Figs. 9 and 10). Markedly different community structures also developed whether rewetting occurred with sterile stream water or with water containing the natural bacterial community. Thus, the source of water influences the bacterial community composition in desiccated streambed sediments at least within the first 2 weeks of rewetting. The natural rewetting conditions of streams, such as the Breitenbach, are influenced by specific weather and hydrologic situations. For example, rain water enters the stream as overland flow, interflow, or as groundwater entering the stream by diffuse perfusion through the streambed following an increase of the groundwater table in the catchment [65].

The enzyme activities, measured immediately after rewetting using the perfused core system, were similar to the values achieved when the activities were determined using the suspension approach at the final step of sediment desiccation after 13 weeks (Figs. 5 and 11). The activity levels remained stable for at least 6 h after initiating rewetting (data not shown). Notably, the activity values achieved for dry sediments are potential activities (measured at substrate saturation concentration) assessed immediately after rewetting of sediments. Thus, the coincidence between the two series of measurements was not surprising. We also recognized that the real enzyme activity in dry sediments under actual environmental conditions (more or less dry situation) cannot be determined because of fundamental methodological limitations [37].

Initial enzyme activities in wet sediments were achieved for all enzymes after 6 to 10 days of rewetting, except for phosphatase (Fig. 11, Table S7, cf. Fig. 12). For aminopeptidase, the initial activity level in wet sediment was achieved even more rapidly (after 6 days) and further increased more than for the polymeric carbohydrate-degrading enzymes. For these enzymes, the initial levels in unaffected sediments were reached after 10 days and remained stable until the end of the experiment after 14 days (Fig. 11, Table S7).

The development of activity was distinctly different for phosphatase, an enzyme involved in nutrient remobilization (Fig. 11, Table S7). Consistent with previous observations [7], the phosphatase activity started at a high level upon rewetting, similar to the levels measured in unaffected sediment (Fig. 5D), and subsequently decreased after a few days of rewetting to values below those in non-desiccated sediment. Several processes make unbound phosphorus much more available in sediments upon rewetting [33], and consequently, the production of phosphatases is less necessary [7]. These processes include the increased mineralization of cytoplasmic solutes following the death of organisms during the drying process [66], [67] and the rupture of cells and excretion of osmolytes at initial phases of rewetting [23], [31], [68]. When considering the special circumstances for phosphatases, the ecosystem functionality with respect to extracellular enzyme activity can generally be considered restored after 10 to 14 days of rewetting (Fig. 12).

The findings from the PCA analysis on the concurrent developments between enzyme activities and the abundances of total bacterial cells and different taxonomic groups (Fig. 13) were different than those obtained for drying. It confirms the different evaluation of phosphatase compared with all other enzymes on the development of this enzyme upon rewetting. The close relationships between the total number of bacteria determined via SYBR Green staining and enzyme activities (except with phosphatase) confirmed the coincidence of increasing bacterial abundance and increasing EEA. However, increasing EEA could not be assigned to increasing cell numbers for specific taxonomic groups, except with some reservation for *Betaproteobacteria* and a small trend observed for *Alpha*- and *Gammaproteobacteria*. However, the phylum of *Bacteroidetes* did not show any relationship to enzyme activities during rewetting, although *Bacteroidetes* are a relevant group in streambed sediments [30], considerable numbers of these bacteria were observed in the initial wet sediment and they simultaneously decreased with decreasing EEA during desiccation. *Actinobacteria* exhibited an opposite trend compared with enzyme activities (except phosphatase activity). However, we cannot conclude that these bacteria are unable to produce extracellular enzymes but rather that the environmental conditions in streambed sediments are less favorable for their growth.

Thus, it is reasonable to speculate that important bacteria for the production of extracellular enzymes involved in the degradation of polymeric compounds occur particularly within *Betaproteobacteria*. Nevertheless, organisms exhibiting such functions are also common among *Bacteroidetes*. However, this group was not recovered after 2 weeks of rewetting, and it is unknown how long it might take for the restoration of typical amounts of these bacteria within the sediment community.

### Conclusions and Perspectives

The results obtained from the present study on the experimental drying and rewetting of streambed sediment from the Breitenbach suggest that in typical temperate Central European streams, the microbial community is not resistant [69] against disturbance through desiccation occurring for 13 weeks, but uncertainties remain with respect to the resilience of these communities.

During desiccation, the bacterial community composition distinctly shifted toward the composition typical for soils, exhibiting increased proportions of *Actinobacteria* and *Alphaproteobacteria* but decreased proportions of *Bacteroidetes* and *Betaproteobacteria*. This shift is accompanied by decreasing potential activities of extracellular enzymes, most pronounced with aminopeptidases and less pronounced with enzymes involved with the degradation of polymeric carbohydrates. Upon rewetting, the general ecosystem functioning with respect to extracellular enzyme activity is recovered within 10 to 14 days. However, the bacterial community composition does not reach the original composition observed in unaffected sediments after this time, suggesting a lack of resilience in the short term (2 weeks), but functional redundancy of the community [69] with respect to EEA. The current data are limited in determining how much time is needed to achieve the initial natural structure. Nevertheless, it remains unknown whether the community completely recovers or undergoes a permanent change if desiccation events occur more often, more regularly, for longer times and/or at higher temperatures. For example, there is potential for the permanent loss of Gram-negative bacteria, which are more susceptible to osmotic stress when exposed to drying and rewetting [31]. Thus, it is reasonable to speculate that this loss might be followed by the loss of the specialized (“narrow” sensu Schimel [70]) functions of specific groups of Gram-negative bacteria [31], [71].

## Supporting Information

Table S1 Oligonucleotide probes used for bacterial community analysis via CARD-FISH.
(PDF)

Table S2 Sediment chemical characteristics: ranges.
(PDF)

Table S3 Abundances of prokaryotes in experimentally desiccated Breitenbach streambed sediments: ranges.
(PDF)

Table S4 Significant differences between the abundances of investigated prokaryotic groups with respect to desiccation time (weeks) and treatment (fast, intermediate and slow desiccation).
(PDF)

Table S5 Significant differences between extracellular enzyme activities with respect to desiccation time (weeks) and treatment (fast, intermediate and slow desiccation).
(PDF)

Table S6 Abundances of prokaryotes in Breitenbach streambed sediments experimentally rewetted after 13 weeks of desiccation: ranges.
(PDF)

Table S7 Extracellular enzyme activities in Breitenbach streambed sediments experimentally rewetted for 2 weeks after 13 weeks of desiccation.
(PDF)
