# Supplementary material for: Bacterial Community Composition and Extracellular Enzyme Activity in Temperate Streambed Sediment during Drying and Rewetting
Source: PLoS One. 2013 Dec 27;8(12):e83365. doi: 10.1371/journal.pone.0083365 (PMC3873959; doi:10.1371/journal.pone.0083365)
Supplement: Table S1 — Oligonucleotide probes used for bacterial community analysis via CARD-FISH. (PDF) [file pone.0083365.s001.pdf]

Table S1. Oligonucleotide probes used for bacterial community analysis via CARD-FISH.

Taxon	Name	Sequence (5´-3´)	Reference
Bacteria	S-D-Bact-0338-a-A-18	GCTGCCTCCCGTAGGAGT	[1]
Alphaproteobacteria	S-Sc-aProt-0019-a-A-17	CGTTCGYTCTGAGCCAG	[2]
Betaproteobacteria	L-Sc-bProt-1027-a-A-17	GCCTTCCCACTTCGTTT	[2]
Gammaproteobacteria	L-Sc-gProt-1027-a-A-17	GCCTTCCCACATCGTTT	[2]
Bacteroidetes (*Cytophaga-Flavobacteria* group)	S-P-CyFla-0319-a-A-18	TGGTCCGTGTVAGTAG	[3]
Actinobacteria	L-P-Grps-1901-a-A-18	TATAGTTACCACCGCCGT	[4,5]
Firmicutes	LGC354B	CGGAAGATTCCCTACTGC	[6]

The probes were 5‘-aminolinked oligonucleotides labelled with horseradish peroxidase.

References

1. Amann R, Krumholz L, Stahl DA (1990) Fluorescent-oligonucleotide probing of whole cells for determinative, phylogenetic, and environmental studies in microbiology. Journal of Bacteriology 172: 762-770.
2. Manz W, Amann RI, Ludwig W, Wagner M, Schleifer KH (1992) Phylogenetic oligonucleotide probes for the major subclasses of proteobacteria: problems and solutions. Systematic and Applied Microbiology 15: 593-600.
3. Manz W, Wagner M, Amann R, Schleifer KH (1994) In-situ characterization of the microbial consortia active in 2 waste-water treatment plants. Water Research 28: 1715-1723.
4. Berchtold M., Chatzinotas A, Schonhuber W, Brune A, Amann R, et al. (1999) Differential enumeration and in situ localization of microorganisms in the hindgut of the lower termite Mastotermes darwiniensis by hybridization with rRNA-targeted probes. Archives of Microbiology 172: 407-416.
5. Roller C, Wagner M, Amann R, W. Ludwig, Schleifer KH (1994) In-situ probing of gram-positive bacteria with high DNA G+C content using 235-ribosomal-RNA-targeted oligonucleotides. Microbiology-SGM 140: 2849-2858.
6. Meier H, Amann R, Ludwig R, Schleifer K-H (1999) Specific oligonucleotide probes for in situ detection of a major group of gram-positive bacteria with low DNA G+C content. Systematic and Applied Microbiology 186-196.
