# Supplementary material for: Bacterial Community Composition and Extracellular Enzyme Activity in Temperate Streambed Sediment during Drying and Rewetting
Source: PLoS One. 2013 Dec 27;8(12):e83365. doi: 10.1371/journal.pone.0083365 (PMC3873959; doi:10.1371/journal.pone.0083365)
Supplement: Table S2 — Sediment chemical characteristics: ranges. (PDF) [file pone.0083365.s002.pdf]

Table S2. Sediment chemical characteristics: ranges.

	weeks	fast	intermediate	slow
C:N ratio	0		8.0-9.6	
	2	7.5-9.9	8.1-10.7	8.3-10.9
	4	8.3-9.2	8.0-9.8	8.8-9.8
	8	8.5-9.5	8.3-9.9	8.4-13.8
	13	8.0-9.6	8.3-9.1	7.7-10.3
Nitrogen (µg N g dw^{-1})	0		1.41-1.86	
	2	2.16-2.93	2.23-3.15	4.67-12.45*
	4	0.62-2.71	2.38-3.19	4.51-6.55*
	8	2.24-2.85	2.46-2.82	5.63-6.45*
	13	2.47-2.96	2.39-3.01	5.96-7.61*
Nitrate (µg N g dw^{-1})	0		1.03-1.32	
	2	1.96-2.66	1.91-2.89	3.97-11.22
	4	0.29-2.49	2.08-2.95	4.07-6.21*
	8	1.86-2.58	2.16-2.65	5.46-6.26*
	13	2.04-2.34	2.02-2.57	5.71-7.38*
Ammonium (µg N g dw^{-1})	0		0.08-0.62	
	2	0.04-0.21	0.13-0.46	0.10-0.50
	4	0.06-0.21	0.09-0.20	0.12-0.17
	8	0.13-0.23	0.08-0.35	0.07-0.20
	13	0.33-0.55	0.15-0.38	0.15-0.17

Ranges of C:N ratios, concentrations of nitrogen, nitrate, and ammonium in streambed sediments during different desiccation scenarios (n=4-5). The asterisks indicate significant differences between the treatments (ANOVA, * = $P<0.05$).
