# Supplementary material for: Bacterial Community Composition and Extracellular Enzyme Activity in Temperate Streambed Sediment during Drying and Rewetting
Source: PLoS One. 2013 Dec 27;8(12):e83365. doi: 10.1371/journal.pone.0083365 (PMC3873959; doi:10.1371/journal.pone.0083365)
Supplement: Table S3 — Abundances of prokaryotes in experimentally desiccated Breitenbach streambed sediments: ranges. (PDF) [file pone.0083365.s003.pdf]

Table S3. Abundances of prokaryotes in experimentally desiccated Breitenbach streambed sediments: ranges.

	weeks	fast	intermediate	slow
Prokaryotes	0		2.4-6.0	
(10^9 cells mL^{-1})	2	0.8-4.0*	1.5-3.8*	1.1-3.7*
	4	3.1-6.2	2.9-5.3	2.8-6.0
	8	1.7-10.8	4.4-9.1	2.3-7.4
	13	0.6-7.0	6.1±1.4	2.0-9.0
Bacteria	0		1.0-1.5	
(10^9cells mL^{-1})	2	0.7-2.7	0.3-2.3	1.7-2.3
	4	0.8-1.3	0.3-0.8	0.5-1.8
	8	1.5-1.5	1.1-2.3	1.5-3.0
	13	1.6-1.9	1.7-2.7	1.1-1.8
Alphaproteobacteria	0		2.3-18.5	
(10^7 cells mL^{-1})	2	13.2-44.9*	10.3-38.3*	7.0-57.8
	4	11.9-24.8	0.1-58.3	0.4-93.5*
	8	15.6-45.2*	0.5-79.9	16.5-28.7
	13	2.9-26.9	21.0-50.6*	1.5-33.8*
Betaproteobacteria	0		7.8-51.4	
(10^7 cells mL^{-1})	2	4.5-22.6	11.2-19.1	3.6-44.0
	4	5.3-10.8*	3.6-15.6*	15.7-31.5
	8	2.6-21.9	7.2-17.5	3.3-19.5
	13	16.0-19.1	5.7-18.3	0.0-20.0
Gammaproteobacteria	0		0.0-5.5	
(10^7 cells mL^{-1})	2	0.0-7.4	0.0-10.7	0.1-4.6
	4	0.4-9.4	0.0-6.3	0.0-10.2
	8	1.9-5.8	0.1-7.5	0.0-5.3
	13	0.0-2.9	0.0-1.9	1.3-14.1
Bacteroidetes	0		1.4-12.8	
(10^7 cells mL^{-1})	2	0.8-9.4	1.6-6.2	0.4-9.8
	4	0.4-1.9	0.4-13.4	3.5-7.9
	8	0.4-2.1*	0.5-5.7	0.9-4.0
	13	1.4-2.4	1.1-5.7	0.0-4.8
Actinobacteria	0		0.4-5.3	
(10^7 cells mL^{-1})	2	3.8-37.4	4.9-11.9	1.3-6.0
	4	5.9-13.2*	3.5-9.9	4.6-11.2*
	8	8.5-20.5*	17.1-23.4*	31.9-48.0**
	13	10.3-23.9*	5.9-31.4	5.7-32.2*
Firmicutes	0		1.0-10.3	
(10^7 cells mL^{-1})	2	0.0-2.0	0.3-3.1	0.0-3.6
	4	1.9-9.0	0.1-5.9	0.1-3.5
	8	0.0-2.6	0.65-8.3	0.0-8.7
	13	1.5-2.1	0.4-3.5	0.8-3.1

The abundance of prokaryotes was determined after SYBR Green staining whereas the abundances of different taxonomic groups were determined via CARD-FISH (n=4). The asterisks indicate significant differences between wet sediment from day 0 and the treatment samples (ANOVA, * = P<0.05, ** = P< 0.01).
