# Supplementary material for: Bacterial Community Composition and Extracellular Enzyme Activity in Temperate Streambed Sediment during Drying and Rewetting
Source: PLoS One. 2013 Dec 27;8(12):e83365. doi: 10.1371/journal.pone.0083365 (PMC3873959; doi:10.1371/journal.pone.0083365)
Supplement: Table S4 — Significant differences between the abundances of investigated prokaryotic groups with respect to desiccation time (weeks) and treatment (fast, intermediate and slow desiccation). (PDF) [file pone.0083365.s004.pdf]

Table S4. Significant differences between the abundances of investigated prokaryotic groups with respect to desiccation time (weeks) and treatment (fast, intermediate and slow desiccation).

		Fast					Intermediate					Slow				
	Week	0	2	4	8	13	0	2	4	8	13	0	2	4	8	13
Alphaproteobacteria	0	---	*				---	*			*	---	*			
	2	*	---				*	---				*	---			
	4			---					---					---		
	8				---					---					---	
	13					---	*				---					---
Betaproteobacteria	0	---		*			---					---				
	2		---					---	*				---			
	4	*		---				*	---					---		
	8				---					---					---	
	13					---					---					---
Gammaproteobacteria	0	---					---			**	**	---				
	2		---					---					---			
	4			---					---					---		
	8				---		**			---					---	
	13					---	**				---					---
Bacteroidetes	0	---		***	***		---					---		**		
	2		---	*				---		*			---			
	4	***	*	---					---			**		---		
	8	***			---			*		---					---	
	13					---					---					---
Actinobacteria	0	---		*	*	*	---		*			---		*	***	*
	2		---					---					---	*		
	4	*		---			*		---	***		*	*	---		
	8	*			---				***	---		***			---	
	13	*				---					---	*				---
Firmicutes	0	---	*	*			---					---				
	2	*	---					---					---			
	4	*		---					---					---		
	8				---					---					---	
	13					---					---					---

ANOVA, * = P<0.05, ** = P<0.01, *** = P<0.001, empty field = P>0.05, --- not applicable
