# Supplementary material for: Bacterial Community Composition and Extracellular Enzyme Activity in Temperate Streambed Sediment during Drying and Rewetting
Source: PLoS One. 2013 Dec 27;8(12):e83365. doi: 10.1371/journal.pone.0083365 (PMC3873959; doi:10.1371/journal.pone.0083365)
Supplement: Table S5 — Significant differences between extracellular enzyme activities with respect to desiccation time (weeks) and treatment (fast, intermediate and slow desiccation). (PDF) [file pone.0083365.s005.pdf]

Table S5. Significant differences between extracellular enzyme activities with respect to desiccation time (weeks) and treatment (fast, intermediate and slow desiccation).

		Fast					Intermediate					Slow				
	Week	0	2	4	8	13	0	2	4	8	13	0	2	4	8	13
Alpha-glucosidase	0	---		*			---		*	*		---		*		
	2		---	*	*			---					---			
	4	*	*	---			*		---			*		---		
	8		*		---	*	*			---					---	
	13				*	---					---					---
Beta-glucosidase	0	---					---					---				
	2		---	*	*	*		---					---			
	4		*	---					---					---		
	8		*		---					---					---	
	13		*			---					---					---
Beta-xylosidase	0	---					---					---	*		*	*
	2		---		*			---	*	*	*	*	---	*	*	*
	4			---				*	---				*	---	*	*
	8		*		---			*		---		*	*	*	---	
	13					---		*			---	*	*	*		---
Phosphatase	0	---					---					---				
	2		---	*	*			---	*		*		---	*		
	4		*	---		*		*	---	*			*	---		*
	8		*		---				*	---					---	
	13			*		---		*			---			*		---
Aminopeptidase	0	---		*	*	*	---		*	*	*	---	*	*		
	2		---	*				---				*	---	*		
	4	*	*	---			*		---			*	*	---	*	
	8	*			---	*	*			---				*	---	*
	13	*			*	---	*				---				*	---

ANOVA, * = P<0.05, ** = P<0.01, *** = P<0.001, empty field = P>0.05, --- not applicable
