# Supplementary material for: Bacterial Community Composition and Extracellular Enzyme Activity in Temperate Streambed Sediment during Drying and Rewetting
Source: PLoS One. 2013 Dec 27;8(12):e83365. doi: 10.1371/journal.pone.0083365 (PMC3873959; doi:10.1371/journal.pone.0083365)
Supplement: Table S6 — Abundances of prokaryotes in Breitenbach streambed sediments experimentally rewetted after 13 weeks of desiccation: ranges. (PDF) [file pone.0083365.s006.pdf]

Table S6. Abundances of prokaryotes in Breitenbach streambed sediments experimentally rewetted after 13 weeks of desiccation: ranges.

Treatment	Days	Prokaryotes (10^9 cells mL^{-1})	*Bacteria* (10^9 cells mL^{-1})	*Alphaproteo-bacteria* (10^7 cells mL^{-1})	*Betaproteo-bacteria* (10^7 cells mL^{-1})	*Gammaproteo-bacteria* (10^7 cells mL^{-1})	*Bacteroidetes* (10^7 cells mL^{-1})	*Actinobacteria* (10^7 cells mL^{-1})	*Firmicutes* (10^7 cells mL^{-1})
dry	0	0.6-7.0	0.7-2.7	13.2-44.9*	4.5-22.6	0.0-7.4	0.8-9.4	3.8-37.4	0.0-2.0
with cells	1	2.8-5.6	0.4-2.5	13.2-43.4	27.1-68.4*	10.1-19.5*	6.0-30.5	8.0-47.2	0.8-37.8
	2	1.1-4.5	0.7-1.2	19.0-62.7	109.5-114.9**	2.0-30.9	3.3-42.0	1.5-9.0*	1.8-9.9
	3	2.8-4.7	0.3-2.7	14.2-75.4	23.4-268.5*	6.7-30.3*	7.3-82.3*	2.7-17.5	1.5-5.7
	6	2.7-7.8	0.0-5.1	12.3-44.7	152.2-398.9	10.3-44.1	0.4-18.2	4.3-24.1	3.3-9.0
	10	0.6-1.7*	0.6-5.9	87.5-124.1*	22.0-268.8	3.6-19.0	8.5-22.5*	9.5-18.2	1.3-13.8
	14	6.8-8.5*	1.7-3.8	40.3-183.8	56.7-183.8*	4.3-31.5	2.9-17.1	1.5-9.3	10.6-15.1*
without cells	1	1.5-4.9	0.9-2.2	26.6-41.7	0.6-13.2 *	0.6-13.2	4.3-12.1	4.3-12.1	2.5-14.8*
	2	4.0-1.1	0.3-0.9	13.9-100.7	19.4-69.4	1.4-31.8	0.3-9.6	1.4-45.4	6.3-13.3*
	3	1.9-5.9	1.1-1.3	16.3-126.9	72.6-293.4	49.4-23.5*	2.5-24.3	7.0-9.4	8.0-17.3
	6	0.7-4.0	0.1-3.7	5.2-40.8	26.4-115.1	1.1-112.1	5.3-44.7*	6.5-34.8	2.0-18.1
	10	4.8-8.9	0.2-1.6	9.2-55.8	27.7-58.8*	1.4-103.3	6.4-22.5	2.0-7.7*	2.9-11.4
	14	3.3-6.7	0.4-1.4	31.7-97.3	31.5-80.7*	5.3-9.6*	7.0-9.8 *	7.3-5.0*	5.8-8.0

The abundance of prokaryotes was determined after SYBR Green staining whereas the abundances of different taxonomic groups were determined via CARD-FISH (n=4). The asterisks indicate significant differences between dry sediment from day 0 used for rewetting and the treatment samples (ANOVA, * = P<0.05, ** = P< 0.01).
