# Supplementary material for: Bacterial Community Composition and Extracellular Enzyme Activity in Temperate Streambed Sediment during Drying and Rewetting
Source: PLoS One. 2013 Dec 27;8(12):e83365. doi: 10.1371/journal.pone.0083365 (PMC3873959; doi:10.1371/journal.pone.0083365)
Supplement: Table S7 — Extracellular enzyme activities in Breitenbach streambed sediments experimentally rewetted for 2 weeks after 13 weeks of desiccation. (PDF) [file pone.0083365.s007.pdf]

Table S7. Extracellular enzyme activities in Breitenbach streambed sediments experimentally rewetted for 2 weeks after 13 weeks of desiccation.

Time [h]	Alpha-glucosidase	Beta-glucosidase	Beta-xylosidase	Phosphatase	Aminopeptidase
0	0.94±0.05	11.1±0,9	4.8±0.7	33.5±3.2	2.8±0.4
1	1.7±0.5	10.8±0.9	3.8±0.3	38.4±3.1	4.6±0.2
24	1.1±0.2	7.2±0.9*	2.1±0.1	36.8±3.2	11.0±1.0
48	1.8±0.9	11.6±0.9**	3.0±0.3	38.1±1.2	32.9±1.3
72	2.1±0.8	12.9±1.4	3.6±0.6	26.7±7.7	47.9±1.9
144	4.4±1.9	14.5±1.4	5.1±2.3	4.6±3.3*	89.6±4.2
240	12.2±2.3**	33.6±0.7***	13.7±1.4*	2.3±1.0*	126±18*
336	15.2±3.3**	38.7±2.0***	16.8±1.4**	6.2±4.9*	200±14**

The measurements of extracellular enzyme activities were performed immediately after rewetting (day 0) and after 1, 2, 3, 6, 10, and 14 days of perfusion with filtered and boiled Breitenbach stream water. Means (nmol mL^{-1} h^{-1}) with SD are given (n=3). The asterisks indicate significant differences between the treatment samples and dry sediment from day 0 used for rewetting (t-test, * = P<0.05, ** = P<0.01, *** = P<0.001).
